# A Meta-Analysis of Sampled Maximal Aerobic Capacity Data for Boys Aged 11 Years Old or Less Obtained by Cycle Ergometry

**DOI:** 10.3390/life13020276

**Published:** 2023-01-19

**Authors:** Iva Jurov, Jure Demšar, Thomas McCurdy

**Affiliations:** 1 Sports Medicine Centre, Clinical Institute of Occupational, Traffic and Sports Medicine, University Medical Centre Ljubljana, 1000 Ljubljana, Slovenia; 2 Faculty of Computer and Information Science, University of Ljubljana, 1000 Ljubljana, Slovenia; 3 Health and Environmental Impacts Division (HEID), Office of Air Quality Planning and Standards (OAQPS), US EPA, Research Triangle Park, 27709 NC, USA

**Keywords:** maximal oxygen consumption, children, boys, cycle ergometry, aerobic fitness

## Abstract

The aim of this study was to develop distributions of VO_2max_ based on measured values that exist in the literature in prepubertal boys using cycle ergometry. PRISMA guidelines were followed in conducting this research. One database was searched for peak and maximal VO_2_ values in healthy boys with mean age under 11 years old. Data were split into articles reporting absolute and relative VO_2max_ values and analyzed accordingly. Multilevel models grounded in Bayesian principles were used. We investigated associations between VO_2max_ and body mass, year of the study, and country of origin. Differences in “peak” and “maximal” VO_2_ were assessed. Absolute VO_2max_ (Lmin^−1^) increases with age (P ~100%) but mean relative VO_2max_ does not change (P ~100%). Absolute VO_2max_ is higher in more recent studies (P = 95.7 ± 0.3%) and mean relative VO_2max_ is lower (P = 99.6 ± 0.1%). Relative VO_2max_ in the USA is lower compared with boys from other countries (P = 98.8 ± 0.2%), but there are no differences in absolute values. Mean aerobic capacity estimates presented as “peak” values are higher than “maximal” values on an absolute basis (P = 97.5 ± 0.3%) but not on a relative basis (P = 99.6 ± 0.1%). Heavier boys have lower cardiorespiratory fitness (P ≈ 100%), and body mass seems to be increasing faster with age in the USA compared with other countries (P = 92.3 ± 0.3%). New reference values for cardiorespiratory fitness are presented for prepubertal boys obtained with cycle ergometry. This is new, as no reference values have been determined so far based on actual measured values in prepubertal boys. Aerobic capacity normalized to body weight does not change with age. Cardiorespiratory fitness in prepubertal boys is declining, which is associated with increasing body mass over the last few decades. Lastly, this study did not find any statistically significant difference in the sample’s mean aerobic capacity estimates using the ”peak” and “maximum” distinctions identified in the literature.

## 1. Introduction

Cardiopulmonary exercise testing is considered the gold standard for cardiorespiratory fitness (CRF) in pediatric medicine [1]. In exercise testing, maximal oxygen uptake (VO_2max_) is determined by using indirect calorimetry, which requires a skilled clinician and the use of standardized exercise treadmill protocols or cycle ergometry [2]. Reference values for CRF are needed to assess disease progression, for intervention monitoring, or to assess suboptimal aerobic performance [3]. Even though both treadmills and cycle ergometers are considered criterion measures of CRF, the two methods often produce statistically different estimates for the same child. The differences may be as large as 7–15%, with treadmill estimates being higher than cycle estimates [4,5,6]. Cycle ergometry has an advantage, however, as the test is not easily constrained by the mechanical limitations of the patient, such as deviant walking patterns and soreness in joints. In addition, during cycle ergometry, there is a lower chance of movement artifacts in the ECG and blood pressure recordings [1].

Another issue in exercise physiology is which criteria should be used for determining CRF. For a VO_2max_ determination, a plateau of VO_2_ needs to be achieved. Usually, this is hard to attain in children. If relaxed criteria are used, the highest VO_2_ value is then called VO_2peak_. We know that the exercise physiology literature distinguishes between VO_2max_ and VO_2peak_ metrics, and we preserve this terminological difference in the tables but use CRF to designate maximal oxygen consumption in both cases. Differences in the two metrics are another aspect of uncertainty surrounding what is being measured. In fact, some reviewers state that the highly conditional nature of CRF estimates makes their validity and reliability questionable, especially during growth and maturation [7].

The literature has shown that a statistically significant difference in CRF could exist between girls and boys using absolute VO_2max_ values or relative VO_2max_ to body mass, and boys could have higher CRF values [7]. In addition, young girls and boys participate in different activities, and girls are less involved in organized sports and spend less time practicing [8]. These differences in activities cause boys to be outdoors more than girls, on average [9,10]. Thus, there are physiological and behavioral reasons why CRF estimates should be gender specific. Secondly, younger children spend more time outdoors and undertake more moderate and vigorous physical activities than older children, even if that time is modest—not optimal—for almost everyone [8,11,12]. When looking into CRF for children, using only children with mean age under 11 years of age can minimize physiological complications associated with puberty, resulting in significant changes in total body and muscle mass, stroke volume, growth velocity, oxygen uptake kinetics, fat oxidation rates, and blood lactate responses to work [13,14,15].

Given the lack of age- and gender-specific CRF reference values in prepubertal children, there is a need to develop observed distributions of VO_2max_ based on criterion methods rather than estimated or regression-based predicted values that are currently widely used. To the best of our knowledge, this is the first meta-analysis to critically examine CRF in boys 11 y old or less measured with cycle ergometry and distinguishing between VO_2max_ and VO_2peak_ indicators. The aim of this analysis is to provide researchers, medical experts, and sports practitioners with criterion-based observed values based on sampled studies identified in the literature. In addition, the aim of this paper is to assess whether there are significant differences in VO_2max_ and VO_2peak_ in boys under 11 y old and to compare values in girls of the same age.

## 2. Materials and Methods

### 2.1. Design

The Consideration of Population, Intervention, Comparator, Outcomes, and Study design (PICOS) framework was used.

### 2.2. Population

Included subjects were subjects who: (1) had mean age under 11 years old, (2) were stated to be healthy, (3) were without cardiovascular disease, pulmonary diseases (except asthma), morbid obesity, developmental disabilities, or muscular dystrophies, and (4) were free from injury. Overweight participants were included. Children with asthma were also included, as they seemed to have physical activity levels comparable with those of the normal pediatric population [16].

### 2.3. Intervention

Regardless of the interventions reported in many of the original articles, only pre-intervention data were used.

### 2.4. Comparator

VO_2max_ and VO_2peak_ metrics used to denote aerobic capacity were compared. In addition, comparisons were made based on the year and location of the study (USA versus non-USA countries; conducted solely to provide compatible sample sizes).

### 2.5. Outcomes

The main outcomes were VO_2max_ and VO_2peak_ metrics measured with cycle ergometry.

### 2.6. Study Design

Articles were considered for the analysis if: (1) they were published in a peer-reviewed journal, (2) they had mean/standard deviation VO_2max_/VO_2peak_ parameters for each sample, along with mean age data for the subjects, and (3) if maximal effort was achieved during the incremental test.

**Figure 1:**
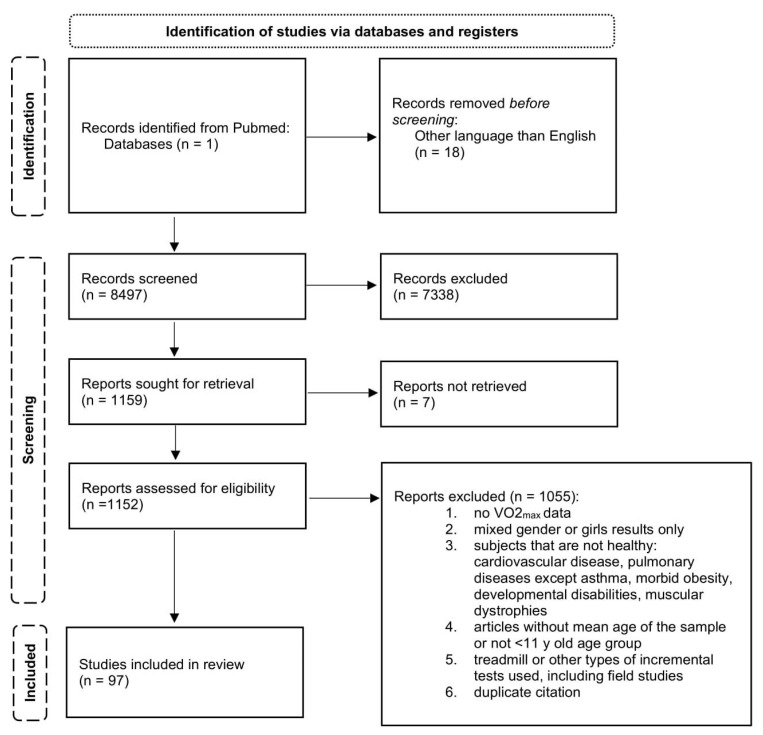
Results of database searches and criteria used in the selection of studies included finding VO_2max_ values in boys under 11 years old.

These measures provided us with children (girls and boys) that used various testing methods for measuring CRF. Consequently, all articles reporting CRF of girls and mixed-gender groups were removed from further analysis. In addition, analysis excluded articles with graphical results only, field studies, treadmill incremental tests, or other nonstandardized protocols and types of incremental tests. The study followed PRISMA guidelines, and the flow diagram presenting the study design can be found in Figure 1.

A systematic electronic literature search was conducted in Pubmed database until 2019 using key search words ((children) AND (oxygen consumption) OR (aerobic power) OR (peak oxygen consumption) OR (VO_2_) OR (VO_2max_) OR (VO_2peak_)). During the first search, potential articles included boys and girls with incremental tests in cycle ergometry and treadmills. All potential articles up to 2019 were hand searched by two researchers. In 2022, the same criteria were used to conduct an additional search from 1 January 2019 to 31 March 2022. Finally, articles that included data from girls and using treadmills were excluded. It is beyond the scope of one manuscript to include boys and girls, so girls were analyzed in a separate paper (in publication). This research includes only the comparison between boys and girls to determine whether there are any differences in cardiorespiratory fitness.

### 2.7. Statistical Analysis

All statistical conclusions developed in this paper utilize applied Bayesian inferential methodology included in the STAN library for R programming [17]. Among other attributes and capabilities, STAN promotes the use of Bayesian inferential models to allow a researcher to evaluate the likelihood that one distribution—in our case, VO_2max_—has the same statistical properties as another distribution purported to describe the same phenomenon. In general, random probability Markov chain Monte Carlo (MCMC) algorithms are used to sample from the two distributions to facilitate the comparisons. A wide variety of Bayesian model comparison techniques are available in STAN to facilitate these types of statistical testing.

When comparing two groups, we used a simple normal model (the so-called Bayesian *t*-test):

y ~ N(μ,σ),

where *y* is the input data (VO_2max_ or VO_2peak_ measurements), *µ* is the location parameter, and *σ* the scale parameter. The default Stan priors (flat improper priors) were used. As observed, *y* is assumed to be approximately normally distributed.

For the linear regression model, the equation:

(1) $$y\;\sim \;N\left(\alpha_{1}+\beta_{1}x,\;\alpha_{2}+\beta_{2}x\right),$$

was used, where *y* is the input data (VO_2max_ or VO_2peak_ measurements), *x* is the dependent variable (e.g., age or year of study), *α*_1_ is the intercept for the location parameter, *β*_1_ is the regression coefficient for the location parameter, *α*_2_ is the intercept for the scale parameter, and *β*_2_ is the regression coefficient for the scale parameter.

In other words, this model can detect both changes in the mean VO_2max_ and the VO_2max_ between-study variance through time or with age. Before making any statistical inferences, we executed all the necessary diagnostics (e.g., trace plots, estimated sample sizes, posterior predictive checks) to ensure the suitability of our models.

With *P*, we denote the probability that a particular research claim is true. We used a capital *P* to not confuse the probabilities calculated with Bayesian analyses with *P*-values from frequentist statistics. Unlike with *P*-values, with Bayesian statistics, we can directly quantify the probability (*P*) of a particular research question, which arguably provides us with the most direct, transparent, and intuitive measure of how certain we are about a claim we are making. Note that with Bayesian approaches, we can easily calculate the probability that the opposite of a particular claim is true (1 − *P*). Because of all this, the use of Bayesian statistical analyses has been on the rise over the last couple of years [17,18,19]. Uncertainty in all our analyses is reported with the Monte Carlo standard error (MCSE) measure.

## 3. Results

The analyses included 95 study samples that reported absolute values of aerobic capacity (both VO_2max_ and VO_2peak_ metrics) in units of Lmin^−1^ (included articles can be found in Table S1) and 118 study samples that reported relative VO_2max_/VO_2peak_ measures in units of mLkg^−1^min^−1^ (included articles can be found in Table S2) [15,20,21,22,23,24,25,26,27,28,29,30,31,32,33,34,35,36,37,38,39,40,41,42,43,44,45,46,47,48,49,50,51,52,53,54,55,56,57,58,59,60,61,62,63,64,65,66,67,68,69,70,71,72,73,74,75,76,77,78,79,80,81,82,83,84,85,86,87,88,89,90,91,92,93,94,95,96,97,98,99,100,101,102,103,104,105,106,107,108,109,110,111,112,113,114,115]. Observed distributions of VO_2max/peak_ are presented in Table 1.

**Figure 2:**
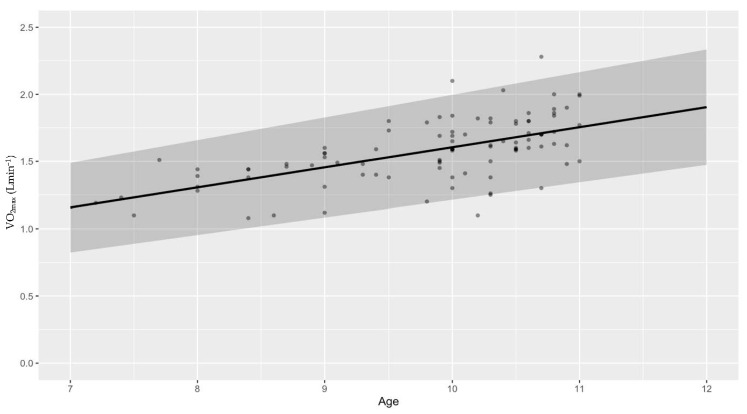

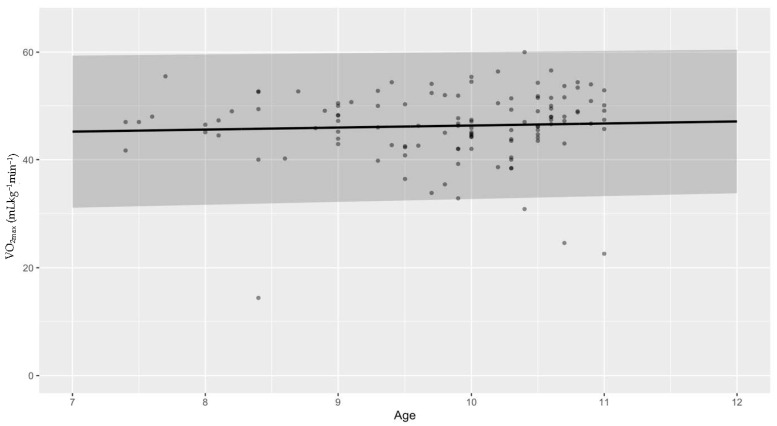
Mean absolute VO_2max_ (Lmin^−1^) increases with age (**upper** figure) in contrast to mean relative VO_2max_ (mLkg^−1^min^−1^) (**lower** figure) and its standard deviation, which do not change with age in boys under 11 years old.

**Figure 3:**
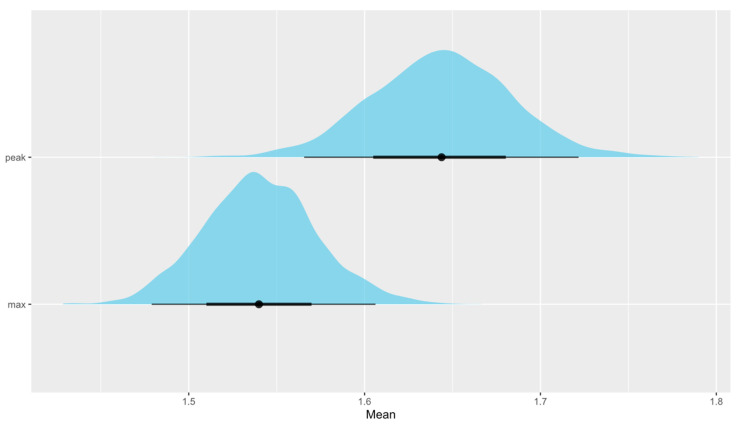

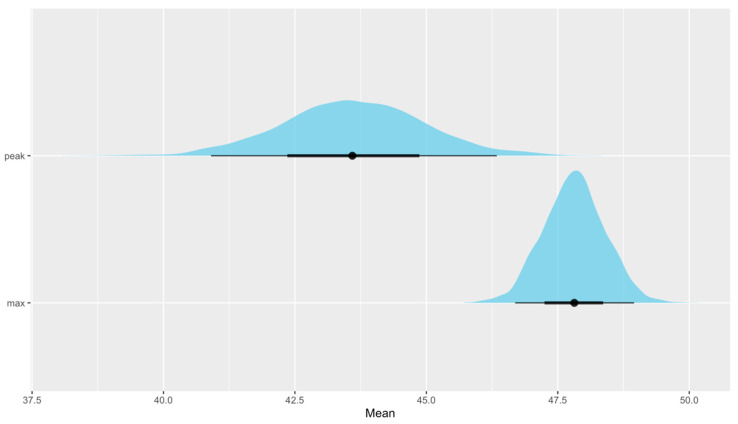
The distribution of mean absolute peak VO_2_ values—VO_2peak_ (Lmin^−1^)—and maximal VO_2_ values—VO_2max_ (Lmin^−1^)—shows that mean VO_2peak_ is higher (**upper** figure). However, mean relative VO_2peak_ (mLkg^−1^min^−1^) is lower than mean relative VO_2max_ (mLkg^−1^min^−1^) (**lower** figure).

**Figure 4:**
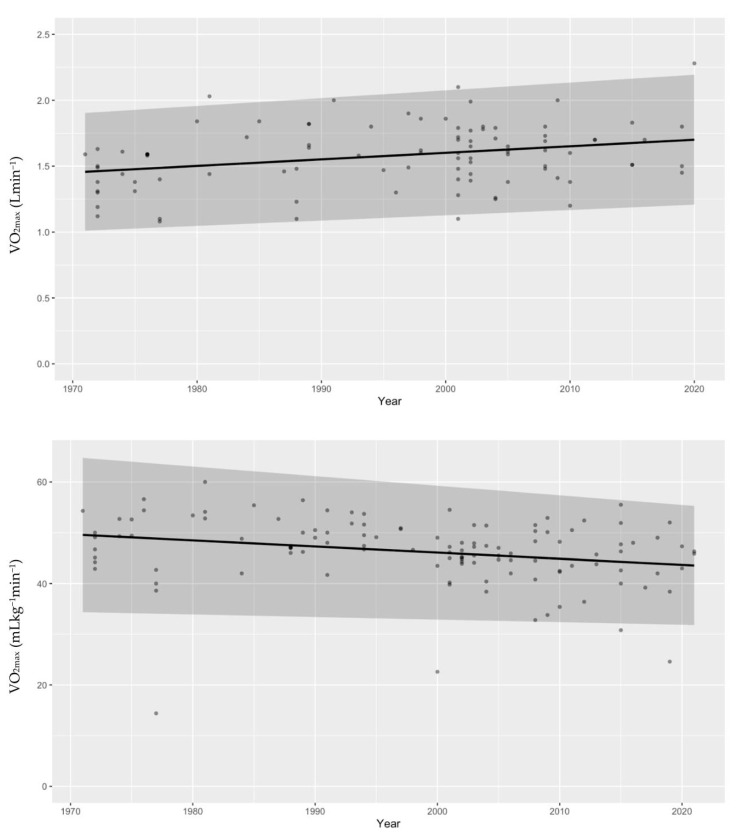
The distribution of mean VO_2max_ during the years shows that more recent studies have higher values of absolute VO_2max_ (Lmin^−1^) (**upper** figure). This is the opposite of the finding with mean relative VO_2max_ (mLkg^−1^min^−1^), which is lower in newer studies (**lower** figure). We can claim this with a probability of 99.57 ± 0.05%. The between-study variability seems to be dropping, and we can claim this with a probability of 90.94 ± 0.3%.

**Figure 5:**
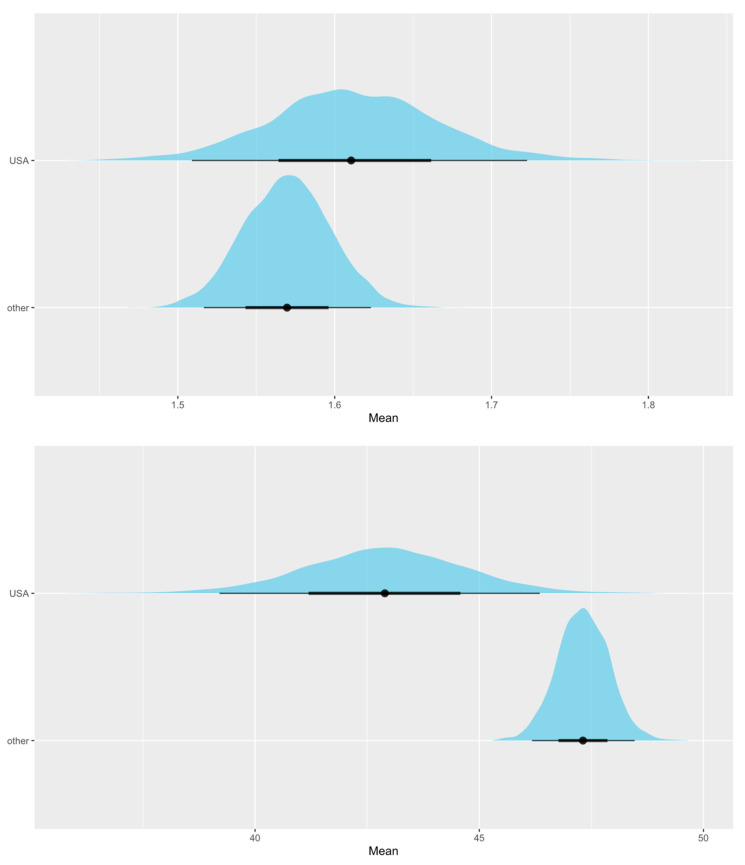
There are no differences in absolute VO_2max_ (Lmin^−1^) between studies with subjects from USA and other countries (**upper** figure), but looking into studies using relative values, higher VO_2max_ (mLkg^−1^min^−1^) values were reported in subjects from other countries than in USA (**lower** figure). We can claim this with a probability of 98.75 ± 0.2%.

**Figure 6:**
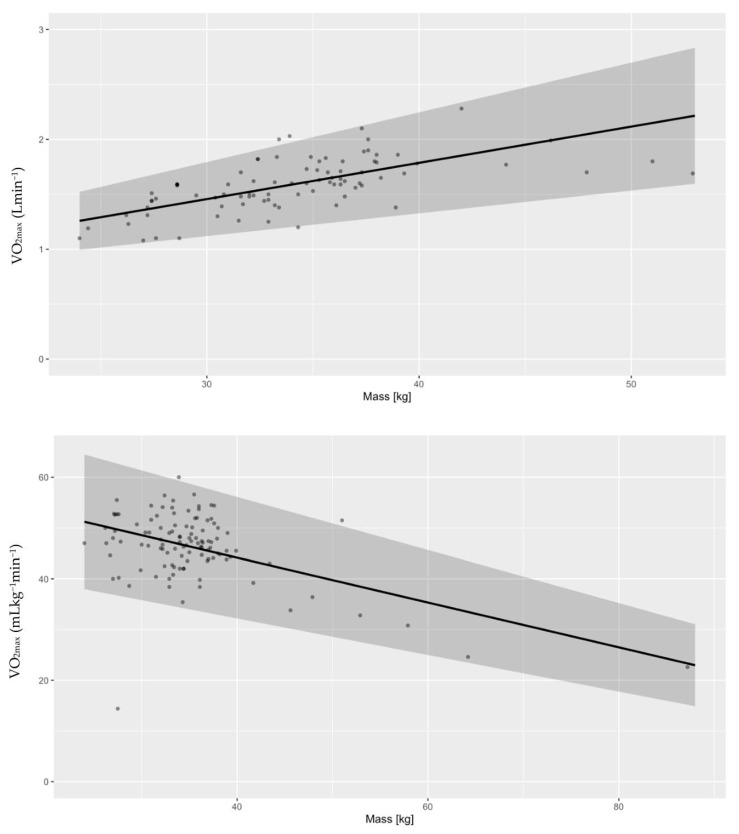
Absolute values of VO_2max_ (Lmin^−1^) are higher in boys with greater body mass (*P* ≈ 100%) (**upper** figure), whereas mean relative VO_2max_ (mLkg^−1^min^−1^) is lower when participants have higher body mass (*P* ≈ 100%) (**lower** figure).

**Figure 7:**
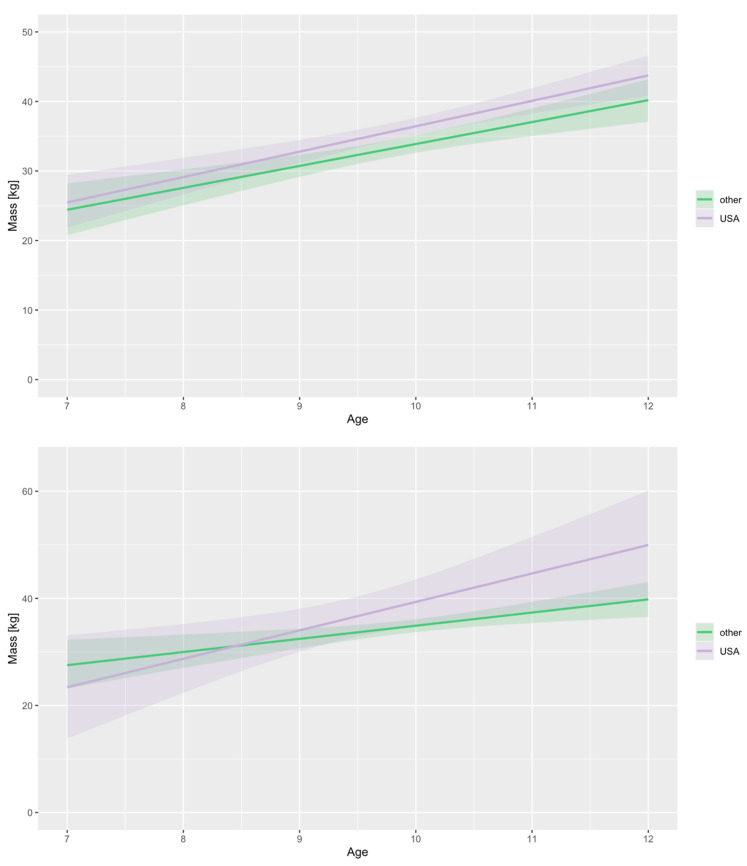
There are no significant differences between trends in articles reporting absolute VO_2max_ values (Lmin^−1^) comparing USA and other countries (**upper** figure) as opposed to articles reporting relative VO_2max_ values (mLkg^−1^min^−1^) (**lower** figure). Analysis showed that in those articles’ bodies: mLkg^−1^min^−1^, relative cardiorespiratory fitness.

We are as sure as we can be (*P* ~100%) that absolute CRF increases with age. The probability that the between-study standard deviation increases with age is 87.1 ± 1.4% (Figure 2). Looking into differences between VO_2max_ and VO_2peak_, this study suggests that mean VO_2peak_ is larger than mean VO_2max_. We can claim this with a probability of 97.5 ± 0.3% (Figure 3). When checking for any changes across the years, the analysis showed that the mean absolute CRF is higher in more recent studies. We can claim this with a probability of 95.7 ± 0.3%. The between-study variability seems to be dropping, but we can claim this only with less than a 90% certainty (*P* = 89.9 ± 0.3%) (Figure 4). We also looked for any differences between CRF in the USA and other countries of the world. We cannot claim there are differences here (Figure 5). Lastly, we looked into body mass. In articles reporting absolute values, CRF (Lmin^−1^) is higher in boys with greater body mass (*P* ≈ 100%) (Figure 6). Looking into differences in body mass between the USA and countries in the rest of the world, there are no significant differences between trends in body mass (Figure 7), but in general, USA boys seem to be heavier (*P* = 96.05 ± 0.4%). What is more, boys in studies using VO_2peak_ seem to be heavier than those with VO_2max_ (*P* = 98.7 ± 0.2%).

We cannot claim that the mean relative CRF or its standard deviation changes with age (Figure 2). Secondly, we checked for differences between VO_2peak_ and VO_2max_. The opposite was found in the absolute CRF. Our study suggests that mean VO_2max_ is higher than mean VO_2peak_. We can claim this with a probability of 99.6 ± 0.1% (Figure 3). Moreover, it seems that the mean relative CRF is lower in more recent studies. We can claim this with a probability of 99.6 ± 0.1%. The between-study variability seems to be dropping, which we can claim with a probability of only 90.9 ± 0.3% (Figure 4). Our study also suggests that the mean relative CRF in other countries is higher than in the USA. We can claim this with a probability of 98.8 ± 0.2% (Figure 5). Investigating body mass in articles reporting relative values, mean relative CRF (mLkg^−1^min^−1^) is lower when participants have higher body mass (*P* ≈ 100%) (Figure 6). It could be that USA boys are a bit heavier on average, but we cannot claim this with a very high probability (*P* = 82.9 ± 0.7%). We did, however, observe that in these articles, body mass seems to be increasing faster with age in the USA compared to other countries (*P* = 92.3 ± 0.3%) (Figure 7). Finally, boys in studies using VO_2peak_ also seem to be heavier in articles reporting relative values compared with studies with VO_2max_ (*P* = 99.6 ± 0.1%).

### 3.1. Is There Any Difference between Boys and Girls?

No significant differences exist between relative cardiorespiratory fitness values in prepubertal boys and girls. The probability that boys have lower values than girls is only 73.6 ± 1%.

### 3.2. Models in Practice

At https://demsarjure.shinyapps.io/vo_2max_/, (access date: 26 December 2022) a simple app can be found in which the measured VO_2max_ in Lmin^−1^, participant’s age, and weight are put in the calculator. The app then uses the fitted Bayesian models to calculate and visualize the percentile for the data that were provided. The app calculates absolute VO_2max_ when VO_2max_ and age are provided and relative VO_2max_ when weight is provided as well. The dashed vertical lines denote the 95% CI.

## 4. Discussion

To the best of our knowledge, this is a meta-analysis with the largest dataset of CRF measurements in boys with mean age under 11 that performed cycle ergometry. Based on the articles included, normative values for prepubertal boys are presented, and a prediction model based on age has been developed for researchers and clinicians to use.

Children with mean ages 4 to 11 were included in this meta-analysis. Relative CRF (normalized to body mass) or its standard deviation did not change with age, which is in line with norms found in boys from 8 to 18 years old [116]. However, articles reporting CRF not normalized to body mass (mean absolute CRF) showed that CRF and its standard deviation in prepubertal boys are dependent on age. This can be explained by increasing body mass as boys age. Body mass is metabolically active tissue that uses oxygen consumption during exercise. This finding is supported by higher absolute CRF in heavier boys in this meta-analysis. Interestingly, mean relative CRF is lower in heavier boys, which we suggest can be explained by the methodology used. We excluded only morbid obesity, so overweight subjects were included in our analysis. The decision to include them seemed necessary since obesity has become a global epidemic during the last three decades, especially in developed countries. In 2013, 23.8% of boys were overweight or obese [117]. In children with obesity, CRF has declined in the last decades, and it is vital to improve the level of physical activity and to improve their aerobic fitness [118]. Creating normative values for boys with normal body mass index only would not be useful for those who are overweight or obese. Heavier children might be more susceptible to cardiovascular risk later in life and will need clinical evaluation and follow-up. To conclude, heavier boys seem to have lower aerobic capacity, which can be explained by overweight individuals also included in this analysis. Having normative values for boys (both absolute and relative CRF values) is, thus, necessary for understanding an individual’s fitness and could gain even greater importance as boys seem to have become heavier in recent years.

Although specifics of determining VO_2peak_ in contrast to VO_2max_ are widely discussed in the literature, there are no recommendations for their use in children based on large studies. This analysis showed that mean VO_2peak_ is higher than mean VO_2max_ in studies reporting absolute values, and the opposite was found in studies with relative values. In theory, lower VO_2peak_ than VO_2max_ could be explained by subjects not reaching their actual maximal oxygen uptake in articles using VO_2peak_ metric, which is a general concern when reporting maximal VO_2_ in children and adults. However, there is no clear explanation why children with measured absolute values would show higher VO_2peak_ than mean VO_2max_. Our study cannot provide clarification of this finding. However, we would like to suggest that higher body mass in boys with VO_2peak_ as compared with boys with VO_2max_ could be a reason for this. We also observed higher body mass in studies using relative VO_2peak_ as compared with boys with relative VO_2max_, but these values are normalized to body mass.

In more recent studies, absolute CRF values (not normalized to body mass) are higher and mean relative CRF values (normalized to body mass) are lower. We can assume this is the result of increasing body mass in boys involved in the studies analyzed. The between-study variability is dropping, which we suggest can be explained by improved methodological approaches and more articles in recent years (85 groups prior to 1995 vs. 128 subject groups after 1995). Understanding that based on these results, prepubertal boys are becoming less fit, which is not only observed or estimated but can now be supported by actual VO_2max_ measurements as well.

Finally, there is no difference between absolute CRF in the USA and other countries, but relative values are lower in the USA. We found an association between higher body mass and higher absolute values, and we can also say with certainty that USA boys are heavier in the studies included in our analysis. Both absolute and relative values thus indicate that prepubertal boys from the USA have lower endurance capacity than boys from other countries. If we try to interpret that with the data from our analysis that body mass in the USA increases faster than in other countries, we can expect endurance capacity to decrease even further in the future. This is alarming since CRF is the most important marker of health among the health-related physical fitness components in children and adolescents [119,120,121], and there is an inverse relationship between cardiorespiratory fitness during childhood and cardiovascular disease risk factors in adulthood [122].

### Limitations

There are some limitations that should be considered. Firstly, we did not distinguish among the many protocols used for each of the approaches during cycle ergometry. These protocols are quite important in estimating aerobic capacity, but protocol nuances used by individual laboratories make sorting them into logical categories very difficult. The same can be said about criteria used to determine whether a child has attained his personal best CRF for a particular test averaging time [123]. These criteria usually include: RER (≥1.0), no change in VO_2_ with increasing workload (i.e., a plateau in VO_2_), visible signs of exhaustion, and attainment of age-predicted heart rate or some percentage of it [5,124] but are not identical in all studies involved.

## 5. Conclusions

New reference values for CRF are presented for prepubertal boys that can be used for physical fitness classification on an individual level for medical experts and sports practitioners. Aerobic capacity normalized to body weight does not change with mean age in boys 4–11 years old, which can be very useful in clinical settings for early diagnosis of reduced cardiorespiratory fitness. It seems that values are not different from those in prepubertal girls.

CRF in prepubertal boys is declining. Our results show that this is associated with increasing body mass, but it also suggests boys with mean age under 11 years old might be less active than in the past. In addition, aerobic capacity is lower in boys in the USA, which is associated with increased body mass. In light of the obesity pandemic, these results indicate more action is needed to improve physical activity in prepubertal boys in order to reduce the likelihood of increased cardiovascular risk later in life. CRF references presented by this analysis can aid in evaluating obesity criteria and physical fitness in prepubertal boys.

Finally, this study did not find any advantage in determining CRF values with the VO_2peak_ or VO_2max_ metrics. Based on our findings, it seems that in prepubertal boys, the differences are not significant enough to be important. This can help researchers and clinicians as they perform cardiopulmonary tests on prepuberal boys.

## Figures and Tables

**Table 1:** CRF values based on sampled studies for boys under 11 years old obtained with cycle ergometry (mean ± SD).

Age (Years)	4–5	5–6	6–7	7–8	8–9	9–10	10–11
VO_2max/peak_ (mLkg^−1^min^−1^)	44.29 ± 7.28	44.67 ± 7.20	45.04 ± 7.12	45.41 ± 7.04	45.79 ± 6.96	46.16 ± 6.88	46.54 ± 6.80
Body mass (kg)	16.43 ± 3.58	19.96 ± 4.16	23.50 ± 4.75	27.03 ± 5.34	30.56 ± 5.93	34.09 ± 6.51	37.62 ± 7.10
VO_2max/peak_ (Lmin^−1^)	0.78 ± 0.14	0.93 ± 0.15	1.08 ± 0.16	1.23 ± 0.17	1.38 ± 0.18	1.53 ± 0.19	1.68 ± 0.20
Body mass (kg)	16.37 ± 3.01	19.68 ± 3.24	22.99 ± 3.47	26.31 ± 3.71	29.62 ± 3.94	32.93 ± 4.18	36.24 ± 4.41

Lmin^−1^, absolute cardiorespiratory fitness.

## Data Availability

All data are available in the Supplementary Materials.

## Supplementary Materials

The following supporting information can be downloaded at: https://www.mdpi.com/article/10.3390/life13020276/s1, Table S1: Included Studies for Absolute VO_2max/peak_ values (Lmin^−1^); Table S2: Included Studies for Relative VO_2max/peak_ values (Lmin^−1^)
