# Supplementary material for: A Meta-Analysis of Sampled Maximal Aerobic Capacity Data for Boys Aged 11 Years Old or Less Obtained by Cycle Ergometry
Source: Life (Basel). 2023 Jan 19;13(2):276. doi: 10.3390/life13020276 (PMC9967809; doi:10.3390/life13020276)
Supplement: Supplementary file 1 [file life-13-00276-s001.zip › Supplementary 1.pdf]

Supplementary file S1

Table S1: Included Studies for Absolute $VO_{2max/peak}$ values ($Lmin^{-1}$)

Age Mean (y)	Age SD (y)	Age range	Body Mass Mean (kg)	Body Mass SD (kg)	Height Mean (cm)	Height SD (cm)	BMI Mean (kg/ m²)	BMI SD (kg/ m²)	VO_{2max} Mean ($Lmin^{-1}$)	VO_{2max} SD ($Lmin^{-1}$)	COV (%)	n	Additional Comments	Peak (P) or max (M) VO_2	Location	Reference
7.5	0.8	7-12	24.0	2.3	126	6	NP	NP	1.10	0.19	17.3	22	BSA<1.0 m³	M	USA	Washington et al. (1988) (1)
8	NP	8	32.7	6.6	133	5.0	18.3	2.8	1.44	0.31	21.5		African-American	M	USA	McMurray et al. (2002) (2)
8	NP	8	30.7	6.0	132	6.0	17.5	2.6	1.39	0.23	16.5		Caucasian	M	USA	McMurray et al. (2002) (2)
8.4	1.3	NP	27.0	4.7	128	6.6	NP	NP	1.08	0.24	22.2	13	Normal, untrained sample	P	USA	Sockolov et al. (1977) (3)
8.6	2.0	NP	27.6	6.9	132	11.3	NP	NP	1.10	0.30	27.3	6	VO2max plateau group	M	PR	Rivera-Brown et al. (2001) (4)
8.9	0.6	7-9	30.4	5.2	132	4.7	17.4	2.4	1.47	0.17	11.6	23		M	USA	Turley et al. (1995) (5)
9	NP	9	37.0	10.0	139	7.0	19.0	3.7	1.56	0.35	22.4		African-American	M	USA	McMurray et al. (2002) (2)
9	NP	9	35.0	8.6	137	6.0	18.5	3.5	1.53	0.27	17.6		Caucasian	M	USA	McMurray et al. (2002) (2)
9.1	0.7	7-9	29.5	4.3	134	6.3	16.2	1.50	1.49	0.20	13.4	12	Cycle ergometer v. treadmill study	M	USA	Turley & Wilmore (1997) (6)
9.3	1.5	7-12	32.0	3.0	139	7.0	NP	NP	1.48	0.24	16.2	30	BSA: 1.0-1.19 m³	M	USA	Washington et al. (1988) (1)
9.3	1.3	NP	36.1	9.1	136	8.9	NP	NP	1.40	0.30	21.4	12	No VO_{2max} plateau	M	PR	Rivera-Brown et al. (2001) (4)
9.4	1.7	7-12	33.2	7.3	139	10.3	NP	NP	1.40	0.25	17.9	32		P	USA	Gilliam et al. (1977) (7)
9.8	0.6	8.2-11.0	34.3	7.8	140	5.5	17.5	3.7	1.20	0.20	16.7	20	VO_{2max} range: 0.6-1.4	M	USA	Swain et al. (2010) (8)
9.8	1.6	8-12	38.0	2.1	143	7.6	NP	NP	1.79	0.21	11.7	12	A priori average VO_{2max}	P	USA	Robertson et al. (2001) (9)
10	NP	10	39.3	9.0	142	7.0	19.3	3.2	1.69	0.29	17.2		African-American	M	USA	McMurray et al. (2002) (2)

10	NP	10	38.2	9.6	141	7.0	19.0	3.8	1.65	0.34	20.6		Caucasian	M	USA	McMurray et al. (2002) (2)
10.0	1.7	8-12	37.3	1.7	143	9.9	NP	NP	2.10	0.17	8.1	12	A priori above average VO_{2max}	P	USA	Robertson et al. (2001) (9)
10.5	1.0	NP	39.9	8.0	143	6.8	NP	NP	1.78	0.25	14.0	11		P	USA	Mahon et al. (2003) (10)
10.5	1.3	NP	37.3	7.3	NP	NP	NP	NP	1.58	0.10	6.3	18		M	USA	Rowland et al. (1993) (11)
10.6	1	NP	38.0	10.0	143	7.0	18.0	3.0	1.86	0.30	16.1	61	Year 1 of a longitudinal study	P	USA	Janz et al. (1998) (12)
10.6	0.2	NP	36.4	1.8	144	20.0	NP	NP	1.80	0.09	5.0	10		M	USA	Del Corral et al. (1994) (13)
10.8	1.0	NP	39.0	11.0	144	7.0	NP	NP	1.86	0.30	16.1	63		M	USA	Janz et al. (2000) (14)
10.9	1.3	NP	37.6	3.4	145	5.0	NP	NP	1.90	0.34	17.9	15		P	USA	Rowland (1997) (15)
10.9	1.1	Total sample only	36.5	8.6	143	7.4	NP	NP	1.48	0.22	14.9	5	ADHD patients	P	USA	Mahon et al. (2008) (16)
10.9	1.1	Total sample only	36.5	8.6	143	7.4	NP	NP	1.62	0.26	16.0	9	ADHD patients	P	USA	Mahon et al. (2008) (16)
11	NP	11	46.2	12.4	149	9.0	20.5	4.0	1.99	0.51	25.6		African-American	M	USA	McMurray et al. (2002) (2)
11	NP	11	44.1	12.6	147	7.0	20.2	4.4	1.77	0.36	20.3		Caucasian	M	USA	McMurray et al. (2002) (2)
7.4	0.3	7	26.3	4.4	126	5.1	NP	NP	1.23	0.28	22.8	18	Test of a field procedure	M	Fra	Van Praagh et al. (1988) (17)
7.7	0.3		27.4	3.4	130	4.4	NP	NP	1.51	0.20	13.2	18		M	Fin	Tompuri et al. (2015) (18)
8.7	NP	8.3-9.8	27.6	3.6	132	6.1	NP	NP	1.46	0.19	13.0	20		M	Swe	Sunnegardh & Bratteby (1987) (19)
9	NP	9	34.0	6.5	140	6.3	17.3	2.5	1.60	0.20	12.5	602	Population-based sample	P	Nor	Kolle et al. (2010) (20)
9	NP	8-10	26.2	2.8	130	4.4	NP	NP	1.31	0.17	13.0	19	Used IBP Handbook (1969)	M	Jap	Ikai & Kitagawa (1972) (21)
9.4	0.3	9-14	31.0	4.5	137	4.8	NP	NP	1.59	0.24	15.1	29	Longitudinal study; 2nd year	M	Nor	Andersen et al. (1976) (22)
9.5	0.3	9	33.4	6.2	139	6.0	17.2	2.4	1.38	0.20	14.5	269	European Youth Heart Study	M	Eur	Ortega et al. (2010) (23)
9.5	0.5	NP	35.3	7.4	142	6.0	NP	NP	1.80	0.30	16.7	177	20% overweight or obese	P	Fin	Agbaje et al. (2019) (24)
9.9	0.4	NP	32.2	3.4	141	4.6	NP	NP	1.49	0.21	14.1	28		M	Nor	Andersen & Ghesquiere (1972) (25)
9.9	0.3	9-10	32.9	5.0	138	6.0	17.1	1.9	1.45	0.25	17.2	53		P	UK	Farr et al. (2019) (26)
9.9	NP	9.5-10.1	35.6	NP	142	11.2	NP	NP	1.83	0.51	27.9	71	Part of the PANIC study	P	Fin	Lintu et al. (2015) (27)

9.9	0.3	NP	32.0	4.0	138	5.0	NP	NP	1.51	0.23	15.2	31		P	UK	McNarry et al. (2015) (28)
10	NP	10	33.3	6.6	139	7.4	NP	NP	1.84	0.31	16.8	20		M	Can	Massicotte et al. (1985) (29)
10	NP	10	NP	NP	NP	NP	NP	NP	1.72	0.27	15.7	26		M	Slo	Dzurenkova et al. (2001) (30)
10	NP	10	NP	NP	NP	NP	NP	NP	1.30	0.20	15.4	15	Rural boys	M	Jap	Yoshizawa (1972) (31)
10.3	0.8	9-12	31.5	3.1	140	5.5	16.0	1.1	1.26	0.18	14.3	20	One obese parent	P	Isr	Weinstein et al. (2004) (32)
10.3	0.8	9-12	32.9	5.4	141	6.2	16.2	1.3	1.25	0.18	14.4	20	Normal-weight parents	P	Isr	Weinstein et al. (2004) (32)
10.3	2.4	NP	34.3	2.2	142	6.2	16.9	1.4	1.50	0.50	33.3	23	Normal-weight boys	P	Can	Peralta-Huertas et al. (2008) (33)
10.4	NP	10	33.9	5.0	141	5.0	NP	NP	2.03	0.30	14.8	31	Year 3 of a longitudinal study	M	Nor	Rutenfranz et al. (1981) (34)
10.5	2.6	NP	51.0	9.0	146	5.2	24.0	3.4	1.80	0.37	20.6	23	Overweight boys	P	Can	Peralta-Huertas et al. (2008) (33)
10.5	0.4	NP	36.3	7.2	140	4.0	NP	NP	1.59	0.20	12.6	15	Control group	P	H-K	McManus (2005) (35)
10.5	NP	10	36.0	NP	NP	NP	NP	NP	1.59	NP		20	VO_{2max} range=1.3-2.0 L/min	M	Nor	Hermansen & Oseid (1971) (36)
10.5	0.5	10-11	37.2	6.6	141	4.7	NP	NP	1.60	0.20	12.5	50	Control group	M	Fra	Mandigout et al. (2001) (37)
10.6	0.3	NP	37.9	5.4	144	4.6	NP	NP	1.80	0.28	15.6	17		M	Fra	Vinet et al. (2003) (38)
10.6	NP	NP	NP	NP	NP	NP	NP	NP	1.66	0.14	8.4	20	Asthmatics (various severity)	M	Swe	Friberg et al. (1989) (39)
10.6	0.3	NP	36.3	6.2	143	7.0	NP	NP	1.71	0.24	14.0	25		P	UK	Fawkner & Armstrong (2004) (40)
10.7	0.4	10-11	37.3	7.5	143	6.1	NP	NP	1.70	0.30	17.6	35	Training group (pre)	M	Fra	Mandigout et al. (2001) (37)
10.7	0.8	NP	30.5	4.2	132	5.8	17.6	2.2	1.30	0.10	7.7	30	Low altitude, low SES	M	Bol	de Jonge et al. (1996) (41)
10.8	0.4	NP	34.9	5.2	146	6.0	NP	NP	1.84	0.24	13.0	45		M	Aut	Gaisl & Buchberger (1980) (42)
10.8	NP	9-10	35.2	5.9	142	6.7	NP	NP	1.72	0.38	22.1	62		M	Can	Cunningham et al. (1984) (43)
10.8	0.5	NP	37.6	7.4	148	7.3	NP	NP	2.00	0.20	10.0	18		M	Can	Docherty & Gaul (1991) (44)
11	NP	10-11	30.8	4.1	135	4.2	NP	NP	1.50	0.26	17.3	18		M	Jap	Ikai & Kitagawa (1972) (21)
8.4	NP	8	27.4	3.9	131	4.5	NP	NP	1.44	0.19	13.2	28	Year 1 of a longitudinal study	M	Nor	Rutenfranz et al. (1981) (34)
8.4	NP	NP	27.4	3.9	131	4.5	NP	NP	1.44	0.19	13.2	29	Part of a longitudinal study	M	Nor	Andersen et al. (1974) (45)
9.9	0.3	9-10	32.9	5.0	1.4	0.1	17.1	1.9	1.5	0.3		53	healthy boys	P	GB	Farr 2019 (26)

10.8	1.7	NP	37.4	12.0	144	11.8	17.5	3	1.89	0.47		18	healthy boys	P	USA	Lu 2019 (46)
7.2	0.6	NP	24.4	3.0	123	5.8	NP	NP	1.19	0.25	21.0	8		M	UK	Davies et al. (1972) (47)
8	NP	8	NP	NP	NP	NP	NP	NP	1.28	0.61	47.7	2		M	Slo	Dzurenkova et al. (2001) (30)
8	NP	7-9	27.2	3.3	131	5.9	NP	NP	1.31	0.19	14.5	12	Inactive; no warmup before testing	M	Isr	Inbar & Bar-Or (1975) (48)
8.7	1.0	NP	31.6	5.7	135	6.0	NP	NP	1.48	0.23	15.5	7		P	Ger	Dunstheimer et al. (2001) (49)
9	NP	9	NP	NP	NP	NP	NP	NP	1.12	0.20	17.9	8	Rural boys	M	Jap	Yoshizawa (1972) (31)
9	NP	9	NP	NP	NP	NP	NP	NP	1.56	0.28	17.9	8		M	Slo	Dzurenkova et al. (2001) (30)
9.5	0.2	9	34.7	6.2	141	6.3	17.4	2.5	1.73	0.28	16.2	10	Test of new VO_{2peak} equation	P	Ice	Arngrímsson et al. (2008) (50)
9.9	1.6	8-12	52.9	13.9	141	11.0	26.1	3.5	1.69	0.26	15.4	7	Obese subjects; pre-exercise program	M	Ita	Lazzer et al. (2008) (51)
10	NP	10	28.6	4.7	135	12.3	NP	NP	1.59	0.23	14.5	4	Twin study; trained (pre)	M	Can	Weber et al. (1976) (52)
10	NP	10	28.6	4.2	135	11.7	NP	NP	1.59	0.13	8.2	4	Twin study; untrained (pre)	M	Can	Weber et al. (1976) (52)
10	NP	10	NP	NP	NP	NP	NP	NP	1.38	0.20	14.5	5	Urban boys	M	Jap	Yoshizawa (1972) (31)
10	NP	10	28.6	4.7	135	12.3	NP	NP	1.58	0.23	14.6	4	Twin Study: Trained pre-study	M	Can	Weber et al. (1976) (52)
10	NP	10	28.6	4.2	135	11.7	NP	NP	1.59	0.13	8.2	4	Twin Study: Untrained pre-study	M	Can	Weber et al. (1976) (52)
10.1	0.5	NP	31.7	2.7	139	4.0	NP	NP	1.41	0.18	12.8	9	Sample by equal LBM	P	UK	Winsley et al. (2009) (53)
10.1	1.8	NP	31.6	5.4	142	8.3	15.5	0.9	1.70	0.50	29.4	9	Normal weight group	P	Aus	Crisp et al. (2012) (54)
10.2	0.4	9-11	32.4	3.5	142	5.1	NP	NP	1.82	0.22	12.1	10	Second experiment	P	Den	Hansen et al. (1989) (55)
10.2	NP	10	28.7	3.0	132	2.2	NP	NP	1.10	0.17	15.5	8		M	Jap	Yamaji & Miyashita (1977) (56)
10.3	0.5	NP	32.2	5.4	142	9.5	NP	NP	1.62	NP		10	Controls for a training experiment (pre)	P	UK	Tolfrey et al. (1998) (57)
10.3	0.2	8-12	NP	NP	NP	NP	NP	NP	1.79	0.10	5.6	16	Trained: test of exercise-induced hypoxia	M	Fra	Nourry et al. (2004) (58)

10.3	0.1	10-11	38.9	5.6	145	4.0	NP	NP	1.38	0.17	12.3	10	Interval training group (pre-training)	P	H-K	McManus (2005) (35)
10.3	0.1	NP	32.4	3.5	142	5.1	NP	NP	1.82	0.22	12.1	10	Second experiment	P	Den	Hansen et al. (1989) (55)
10.3	NP	NP	33.2	5.7	143	7.8	NP	NP	1.61	0.19	11.8	14		P	Den	Andersen et al. (1974) (45)
10.4	0.4	NP	35.9	7.3	140	4.0	NP	NP	1.65	0.16	9.7	10	Continuous training group (pre-training)	P	H-K	McManus (2005) (35)
10.5	0.3	10-11	36.3	7.9	144	7.1	NP	NP	1.64	0.20	12.2	9	First experiment	M	Den	Hansen et al. (1989) (55)
10.6	0.9	NP	34.7	6.9	143	7.1	NP	NP	1.60	NP		12	Training group (pre-experiment)	P	UK	Tolfrey et al. (1998) (57)
10.7	0.3	10-11	35.8	5.7	143	6.0	NP	NP	1.61	0.23	14.3	9		P	UK	Welsman et al. (2005) (59)
10.7	0.9	NP	35.7	6.6	147	8.4	NP	NP	1.70	0.28	16.5	9		P	Bel	Vandekerckhove et al. (2016) (60)
10.7	NP	NP	47.9	13.8	148	9.1	21.7	4.7	1.70	0.50	29.4	9	Overweight group	P	Aus	Crisp et al. (2012) (61)
10.7	2.1	7-10	42.0	15.0	147	8.0	NP	NP	2.28	0.13	5.7	12	Undergoing leukemia treatment	P	Pol	Kowaluk & Wojniewski (2020) (62)
10.8	0.7	NP	35.3	11.0	140	9.4	NP	NP	1.63	0.39	23.9	9		M	UK	Davies et al. (1972) (47)
11	NP	10-11	33.4	7.2	143	8.4	16.3	2.3	2.00	0.20	10.0	12	Normal hearing boys	M	Pol	Zebrowska & Zwierzchowska (2009) (63)
8.4	NP	7-9	27.2	3.3	131	5.9	NP	NP	1.38	0.20	14.5	12	Non-active in sports	M	Isr	Inbar & Bar-Or (1975) (48)

(Y=years, VO_{2max}=maximal oxygen consumption, NP=not provided, BSA=body surface area, LBM=lean body mass, Fra=France, Fin=Finland, Swe=Sweden, Nor=Norway, Jap=Japan, Eur=Europe, Can=Canada, Slo=Slovenia, Isr=Israel, PR=Puerto Rico, H-K=Hong Kong, Bol=Bolivia, Aut=Austria, Ice=Iceland, Ita=Italy, Aus=Australia, Den=Denmark, Bel=Belgium)

References

1. Washington RL, van Gundy JC, Cohen C, Sondheimer HM, Wolfe RR. Normal aerobic and anaerobic exercise data for North American school-age children. *J Pediatr*. 1988;112(2):223–33.

2. McMurray RG, Harrell JS, Bradley C, Deng S, Bangdiwala SI. Predicted maximal aerobic power inouth is related to gender, ethnicity, and

body composition. *Med Sci Sport Exerc*. 2001;33(5):S31.

3. Sockolov R, Irwin B, Dressendorfer RH, Bernauer EM. Exercise performance in 6 to 11ear old boys with Duchenne muscular dystrophy. *Arch Phys Med Rehabil*. 1977;58(5):195–201.

4. Rivera-Brown AM, Alvarez M, Rodríguez-Santana JR, Benetti PJ. Anaerobic power and achievement of V̇O2 plateau in pre-pubertal boys. *Int J Sports Med*. 2001;22(2):111–5.

5. Turley KR, Rogers DM, Harper KM, Kujawa KI, Wilmore JH. Maximal Treadmill versus Cycle Ergometry Testing in Children: Differences, Reliability, and Variability of Responses. *Pediatr Exerc Sci*. 1995;7(1):49–60.

6. Turley KR, Wilmore JH. Cardiovascular responses to submaximal exercise in 7- to 9-yr-old boys and girls. *Med Sci Sports Exerc*. 1997;29(6):824–32.

7. Gilliam TB, Katch VL, Thorland W, Weltman A. Prevalence of coronary heart disease risk factors in active children, 7 to 12ears of age. *Med Sci Sports Exerc*. 1977;9(1):21–5.

8. Swain KE, Rosenkranz SK, Beckman B, Harms CA. Expiratory flow limitation during exercise in prepubescent boys and girls: prevalence and implications. *J Appl Physiol*. 2010;108(5):1267–74.

9. Robertson RJ, Goss FL, Boer N, et al. OMNI scale perceived exertion at ventilatory breakpoint in children: Response normalized. *Med Sci*

Sports Exerc. 2001;33(11):1946–52.

10. Mahon AD, Anderson CS, Hipp MJ, Hunt KA. Heart Rate Recovery from Submaximal Exercise in Boys and Girls. *Med Sci Sports Exerc*. 2003;35(12):2093–7.

11. Rowland TW, Rambusch JM, Staab JS, Unnithan VB, Siconolfi SF. Accuracy of physical working capacity (PWC170) in estimating aerobic fitness in children. *J Sports Med Phys Fitness*. 1993;33(2):184–8.

12. Janz KF, Burns TL, Witt JD, Mahoney LT. Longitudinal analysis of scaling V̇O2 for differences in body size during puberty: The muscatine study. *Med Sci Sports Exerc*. 1998;30(9):1436–44.

13. Del Corral P, Mahon AD, Duncan GE, Howe CA, Craig BW. The effect of exercise on serum and salivary cortisol in male children. *Med Sci Sports Exerc*. 1994;26(11):1297–301.

14. Janz KF, Dawson JD, Mahoney LT. Tracking physical fitness and physical activity from childhood to adolescence: The Muscatine study. *Med Sci Sports Exerc*. 2000;32(7):1250–7.

15. Rowland TW. *The development of aerobic fitness in children*. London: E & FN Spon.; 1997. 179–189 p.

16. Mahon AD, Stephens BR, Cole AS. Exercise responses in boys with attention deficit/hyperactivity disorder: Effects of stimulant medication. *J Atten Disord*. 2008;12(2):170–6.

17. Van Praagh E, Bedu M, Falgairette G, Fellmann N, Coudert J. Comparaison entre over VO2 max direct et indirect chez l'enfant de 7 et 12 ans. Validation d'une épreuve de terrain. *Sci Sport*. 1988;3(4):327–32.

18. Tompuri T, Lintu N, Savonen K, et al. Measures of cardiorespiratory fitness in relation to measures of body size and composition among children. *Clin Physiol Funct Imaging*. 2015;35(6):469–77.

19. Sunnegårdh J, Bratteby LE. Maximal oxygen uptake, anthropometry and physical activity in a randomly selected sample of 8 and 13ear old children in Sweden. *Eur J Appl Physiol Occup Physiol*. 1987;56(3):266–72.

20. Kolle E, Steene-Johannessen J, Andersen LB, Anderssen SA. Objectively assessed physical activity and aerobic fitness in a population-based sample of Norwegian 9- and 15-year-olds. *Scand J Med Sci Sport* [Internet]. 2010 [cited 2022 Jun 30];20(1) Available from: https://pubmed.ncbi.nlm.nih.gov/19422647/. doi:10.1111/j.1600-0838.2009.00892.x.

21. Ikai M, Kitagawa K. Maximum oxygen uptake of japanese related to sex and age. *Med Sci Sports Exerc*. 1972;4(3):127–31.

22. Lange Andersen K, Seliger V, Rutenfranz J, Skrobak-Kaczynski J. Physical performance capacity of children in Norway - Part IV. The rate of growth in maximal aerobic power and the influence of improved physical education of children in a rural community - population parameters in a rural community. *Eur J Appl Physiol Occup Physiol*. 1976;35(1):49–58.

23. Ortega FB, Ruiz JR, Hurtig-Wennlöf A, et al. Cardiovascular fitness modifies the associations between physical activity and abdominal adiposity in children and adolescents: The Europeanouth heart study. *Br J Sports Med*. 2010;44(4):256–62.

24. Agbaje AO, Haapala EA, Lintu N, et al. Peak oxygen uptake cut-points to identify children at increased cardiometabolic risk – The PANIC Study. *Scand J Med Sci Sport*. 2019;29(1):16–24.

25. Andersen KL, Ghesquiere J. Sex differences in maximal oxygen uptake, heart rate and oxygen pulse at 10 and 14ears in Norwegian children. *Hum Biol*. 1972;44(3):413–31.

26. Farr C, Middlebrooke AR, Armstrong N, et al. Objectively measured aerobic fitness is not related to vascular health outcomes and cardiovascular disease risk in 9-10ear old children. *J Sport Sci Med*. 2019;18(3):513–22.

27. Lintu N, Viitasalo A, Tompuri T, et al. Cardiorespiratory fitness, respiratory function and hemodynamic responses to maximal cycle ergometer exercise test in girls and boys aged 9–11ears: the PANIC Study. *Eur J Appl Physiol*. 2015;115(2):235–43.

28. McNarry MA, Farr C, Middlebrooke A, et al. Aerobic Function and Muscle Deoxygenation Dynamics during Ramp Exercise in Children. *Med Sci Sports Exerc*. 2015;47(9):1877–84.

29. Massicotte DR, Gauthier R, Markon P. Prediction of VO2max from the running performance in children aged 10-17ears. *J Sports Med Phys Fitness*. 1985;25(1–2):10–7.

30. Dzurenkova D, Marcek T, Hajkova M. Specialities of assessment of endurance capabilities in sport active children. *Bratisl Lek Listy*. 2001;102(9):432–3.

31. Yoshizawa S. A comparative study of aerobic work capacity in urban and rural adolescents. *J Hum Ergol (Tokyo)*. 1972;1(1):45–65.

32. Weinstein Y, Kamerman T, Berry E, Falk B. Mechanical Efficiency of Normal-Weight Prepubertal Boys Predisposed to Obesity. *Med Sci Sports Exerc*. 2004;36(4):567–73.

33. Peralta-Huertas J, Livingstone K, Banach A, Klentrou P, O'Leary D. Differences in left ventricular mass between overweight and normal-weight preadolescent children. *Appl Physiol Nutr Metab*. 2008;33(6):1172–80.

34. Rutenfranz J, Andersen KL, Seliger V, Klimmer F, Berndt I, Ruppel M. Maximum aerobic power and body composition during the puberty growth period: Similarities and differences between children of two European countries. *Eur J Pediatr*. 1981;136(2):123–33.

35. McManus AM, Cheng CH, Leung MP, Yung TC, Macfarlane DJ. Improving aerobic power in primary school boys: A comparison of continuous and interval training. *Int J Sports Med*. 2005;26(9):781–6.

36. Hermansen L, Oseid S. Direct and indirect estimation of maximal oxygen uptake in pre-pubertal boys. *Acta Paediatr Scand Suppl*. 1971;217:18–23.

37. S M, AM L, D C, P G, P O. Effect of gender in response to an aerobic training programme in prepubertal children. *Acta Paediatr*. 2001;90(1):9–15.

38. Vinet A, Mandigout S, Nottin S, et al. Influence of Body Composition, Hemoglobin Concentration, and Cardiac Size and Function of

Gender Differences in Maximal Oxygen Uptake in Prepubertal Children. *Chest*. 2003;124(4):1494–9.

39. Friberg S, Bevegård S, Graff-Lonnevig V, Hallbäck I. Asthma from childhood to adulthood-A follow-up study of 20 subjects with special reference to work capacity and pulmonary gas exchange. *J Allergy Clin Immunol*. 1989;84(2):183–90.

40. Fawkner SG, Armstrong N. Sex differences in the oxygen uptake kinetic response to heavy-intensity exercise in prepubertal children. *Eur J Appl Physiol*. 2004;93(1–2):210–6.

41. De Jonge R. Effect of anthropometric characteristics and socio-economic status on physical performances of pre-pubertal children living in Bolivia at low altitude. *Eur J Appl Physiol Occup Physiol*. 1996;74(4):367–74.

42. Gaisl G, Buchberger J. *Determination of the aerobic and anaerobic thresholds of 10-11-year-old boys using blood-gas analysis*. Baltimore: University Park Press; 1980. 93–98 p.

43. Cunningham DA, Paterson DH, Blimkie CJR, Donner AP. Development of cardiorespiratory function in circumpubertal boys: A longitudinal study. *J Appl Physiol Respir Environ Exerc Physiol*. 1984;56(2):302–7.

44. Docherty D, Gaul CA. Relationship of body size, physique, and composition to physical performance inoung boys and girls. *Int J Sports Med*. 1991;12(6):525–32.

45. Lange Andersen K, Seliger V, Rutenfranz J, Mocellin R. Physical performance capacity of children in Norway - Part I. Population

parameters in a rural inland community with regard to maximal aerobic power. *Eur J Appl Physiol Occup Physiol*. 1974;33(3):177–95.

46. Lu KD, Bar-Yoseph R, Radom-Aizik S, Cooper DM. A new approach to estimate aerobic fitness using the NHANES dataset. *Scand J Med Sci Sport*. 2019;29(9):1392–401.

47. Davies CT, Barnes C, Godfrey S. Body composition and maximal exercise performance in children. *Hum Biol*. 1972;44(2):195–214.

48. Inbar O, Bar-Or O. The effects of intermittent warm-up on 7-9ear-old boys. *Eur J Appl Physiol Occup Physiol*. 1975;34(1):81–9.

49. Dunstheimer D, Hebestreit H, Staschen B, Starßburg HM, Jeschke R. Bilateral deficit during short-term, high-intensity cycle ergometry in girls and boys. *Eur J Appl Physiol*. 2001;84(6):557–61.

50. Arngrímsson SÁ, Sveinsson T, Jóhannsson E. Peak oxygen uptake in children: Evaluation of an older prediction method and development of a new one. *Pediatr Exerc Sci*. 2008;20(1):62–73.

51. Lazzer S, Molin M, Stramare D, Facchini S, Francescato MP. Effects of an eight-month weight-control program on body composition and lipid oxidation rate during exercise in obese children. *J Endocrinol Invest*. 2008;31(6):509–14.

52. Weber G, Kartodihardjo W, Klissouras V. Growth and physical training with reference to heredity. *J Appl Physiol*. 1976;40(2):211–5.

53. Winsley RJ, Fulford J, Roberts AC, Welsman JR, Armstrong N. Sex difference in peak oxygen uptake in prepubertal children. *J Sci Med Sport*. 2009;12(6):647–51.

54. Crisp NA, Fournier PA, Licari MK, Braham R, Guelfi KJ. Adding sprints to continuous exercise at the intensity that maximises fat oxidation: Implications for acute energy balance and enjoyment. *Metabolism*. 2012;61(9):1280–8.

55. Hansen HS, Froberg K, Nielsen JR, Hyldebrandt N. A new approach to assessing maximal aerobic power in children: the Odense School Child Study. *Eur J Appl Physiol Occup Physiol*. 1989;58(6):618–24.

56. Yamaji K, Miyashita M. Oxygen transport system during exhaustive exercise in Japanese boys. *Eur J Appl Physiol Occup Physiol*. 1977;36(2):93–9.

57. Tolfrey K, Campbell IG, Batterham AM. Aerobic trainability of prepubertal boys and girls. *Pediatr Excercise Sci*. 1998;10(3):248–63.

58. Nourry C, Fabre C, Bart F, Grosbois JM, Berthoin S, Mucci P. Evidence of Exercise-Induced Arterial Hypoxemia in Prepubescent Trained Children. *Pediatr Res*. 2004;55(4):674–81.

59. Welsh L, Roberts RGD, Kemp JG. Fitness and physical activity in children with asthma. *Sport Med*. 2004;34(13):861–70. [cited 2022 Jun 28] Available from: https://pubmed.ncbi.nlm.nih.gov/15487902/.

60. Vandekerckhove K, Coomans I, Moerman A, De Wolf D, Boone J. Characterizing cerebral and locomotor muscle oxygenation to incremental ramp exercise in healthy children: relationship with pulmonary gas exchange. *Eur J Appl Physiol*. 2016;116(11–12):2345–55.

61. Crisp NA, Guelfi KJ, Licari MK, Braham R, Fournier PA. Does exercise duration affect Fat max in overweight boys? *Eur J Appl Physiol*.

2012;112(7):2557–64.

62. Kowaluk A, Woźniewski M. Peak oxygen uptake and exercise capacity of children undergoing leukemia treatment. *Int J Environ Res Public Health*. 2020;17(23):1–18.

63. Zebrowska A, Zwierzchowska A. Spirometric values and aerobic efficency of children and adolescents with hearing loss. *J Physiol Pharmacol*. 2006;57(SUPPL. 4):443–7.
