# Supplementary material for: A Meta-Analysis of Sampled Maximal Aerobic Capacity Data for Boys Aged 11 Years Old or Less Obtained by Cycle Ergometry
Source: Life (Basel). 2023 Jan 19;13(2):276. doi: 10.3390/life13020276 (PMC9967809; doi:10.3390/life13020276)
Supplement: Supplementary file 1 [file life-13-00276-s001.zip › Supplementary 2.pdf]

Supplementary S2

Table S2: Included Studies for Relative $VO_{2max/peak}$ values ($Lmin^{-1}$)

Age Mean (y)	Age SD (y)	Age	Body Mass Mean (kg)	Body Mass SD (kg)	Height Mean (cm)	Height SD (cm)	BMI Mean (kg/m²)	BMI SD (kg/m²)	VO_{2max} Mean ($Lkg^{-1}min^{-1}$)	VO_{2max} SD ($Lkg^{-1}min^{-1}$)	COV (%)	n	Addditional Comments	Peak (P) or max (M) VO_2	Location	Reference
7.4	0.6	6-10	29.9	5.3	130	11.1	NP	NP	41.7	3.3	7.9	6	Individual data: of VO_{2max}: 34.2-51.0	M	USA	Springer et al. (1991) (1)
7.5	0.8	7-12	24.0	2.3	126	6	NP	NP	47.0	6.0	12.8	22	BSA<1.0 m³	M	USA	Washington et al. (1988) (2)
8	NP	8	32.7	6.6	133	5.0	18.3	2.8	45.1	11.8	26.2	NP	African-American	M	USA	McMurray et al. (2001) (3)
8	NP	8	30.7	6.0	132	6.0	17.5	2.6	46.5	8.9	19.1	NP	Caucasian	M	USA	McMurray et al. (2001) (3)
8.4	1.3	NP	27.5	4.7	125	9.4	NP	NP	14.4	5.4	37.5	13	Have Duchenne muscular dystrophy	P	USA	Sockolov et al. (1977) (4)
8.4	1.3	NP	27.0	4.7	128	6.6	NP	NP	40.0	3.9	9.8	13	Normal, untrained sample	P	USA	Sockolov et al. (1977) (4)
8.6	2.0	NP	27.6	6.9	132	11.3	NP	NP	40.2	5.9	14.7	6	VO_{2max} plateau group	M	PR	Rivera-Brown et al. (2001) (5)
8.9	0.6	7-9	30.4	5.2	132	4.7	17.4	2.4	49.1	6.4	13.0	23	Treadmill v. cycle study	M	USA	Turley et al.(1995) *Pediatr Exer Sci* 7:49-60
9	NP	9	37.0	10.0	139	7.0	19.0	3.7	43.9	10.6	24.1	NP	African-American	M	USA	McMurray et al. (2001) (3)
9	NP	9	35.0	8.6	137	6.0	18.5	3.5	45.2	9.9	21.9	NP	Caucasian	M	USA	McMurray et al. (2001) (3)
9.1	0.7	7-9	29.5	4.3	134	6.3	16.2	1.5	50.7	4.6	9.1	12	Cycle ergometer v. treadmill study	M	USA	Turley & Wilmore (1997) (6)
9.3	1.5	7-12.0	32.0	3.0	139	7	NP	NP	46.0	5.0	10.9	30	BSA: 1.0-1.19 m³	M	USA	Washington et al. (1988) (2)
9.3	1.3	NP	36.1	9.1	136	8.9	NP	NP	39.8	7.3	18.3	12	No VO_{2max} plateau	M	PR	Rivera-Brown et al. (2001) (5)
9.4	1.7	7-12	33.2	7.3	139	10.3	NP	NP	42.7	5.5	12.9	32		P	USA	Gilliam et al. (1977) (7)
9.5	0.7	NP	33.3	4.5	141	6.4	NP	NP	40.8	10.9	26.7	16	Evaluation of two exertion scales	P	USA	Barkley et al. (2008) (8)

9.8	0.6	8.2-11.0	34.3	7.8	140	5.5	17.5	3.7	35.4	7.5	21.2	20	VO_{2max} 20.7-47.3	M	USA	Swain et al. (2010) (9)
9.8	1.6	8-12	38.0	2.1	143	7.6	NP	NP	45.0	2.6	5.8	12	A priori average VO_{2max}	P	USA	Robertson et al. (2001) (10)
10	NP	10	39.3	9.0	142	7.0	19.3	3.2	44.3	9.2	20.8	NP	African-American	M	USA	McMurray et al. (2001) (3)
10	NP	10	38.2	9.6	141	7.0	19.0	3.8	44.8	9.8	21.9	NP	Caucasian	M	USA	McMurray et al. (2001) (3)
10.0	1.7	8-12	37.3	1.7	143	9.9	NP	NP	54.5	2.3	4.2	12	A priori above average VO_{2max}	P	USA	Robertson et al. (2001) (10)
10.0	2.0	6-13	34.0	9.0	138	14.0	NP	NP	42.0	6.0	14.3	37		M	USA	Cooper et al. (1984) (11)
10.3	1.0	NP	36.1	8.0	141	8.0	18.1	2.0	38.4	5.0	13.0	19	Lean subjects	P	USA	Rivas et al. (2019) (12)
10.5	1.0	NP	39.9	8.0	143	6.8	NP	NP	45.5	7.0	15.4	11		P	USA	Mahon et al. (2003) (13)
10.5	1.3	NP	37.3	7.3	NP	NP	NP	NP	51.8	4.3	8.3	18		M	USA	Rowland et al. (1993) (14)
10.6	1	NP	38.0	10.0	143	7.0	18.0	3.0	50.0	9.0	18.0	61	Year 1 of a longitudinal study	P	USA	Janz et al. (1998) (15)
10.6	0.2	NP	36.4	1.8	144	2.0	NP	NP	49.5	1.1	2.2	11		M	USA	Del Corral et al. (1994) (16)
10.7	1	NP	64.2	15.0	148	9.0	28.9	5.0	24.6	5.0	20.3	16	Obese group	P	USA	Rivas et al. (2019) (12)
10.8	1.0	NP	39.0	11.0	144	7.0	NP	NP	49.0	8.0	16.3	63		M	USA	Janz et al. (2000) (17)
10.9	1.3	NP	37.6	3.4	145	5.0	NP	NP	50.9	8.3	16.3	15		P	USA	Rowland (1997) (18)
11	NP	9-12	87.2	21.8	157	11.0	31.8	6.5	22.6	4.5	19.9	15	Healthy obese	P	USA	DeStefano et al. (2000) (19)
7.6	0.4	6-18	27.0	4.0	126	5.0	NP	NP	48.0	5.0	10.4	36		M	Fra	Falgairette et al. (1991) (20)
8.1	1.0	NP	34.2	7.9	132	16.0	NP	NP	44.5	10.9	24.5	46		P	Spa	Guerrero et al. (2008) (21)
8.4	NP	NP	27.4	3.9	131	4.5	NP	NP	52.7	3.9	7.4	29	Part of a longitudinal study	M	Nor	Andersen et al. (1974) (22)
9	NP	9	34.0	6.3	140	6.0	17.2	2.4	48.3	7.0	14.5	487		P	Nor	Steene-Johannessen (2009) (23)
9	NP	9	34.0	6.4	140	6.3	NP	NP	47.2	8.0	16.9	279	Euro Youth Heart Study	M	Eur	Wedderkopp et al. (2003) (24)
9	NP	9	34.0	6.5	140	6.3	17.3	2.5	48.2	7.1	14.7	602	Population-based sample	P	Nor	Kolle et al. (2010) (25)
9	NP	8.2-11.3	33.5	6.7	139	6.3	17.3	2.6	50.5	8.7	17.2	1219	Euro. Youth Heart Study(4 Nations)	P	Eur	Adegboye et al. (2011) (26)
9.3	0.7	9-10	32.0	4.0	137	5.0	NP	NP	50.0	7.0	14.0	27		M	Fra	Falgairette et al. (1991) (20)
9.4	0.3	9-14	31.0	4.5	137	4.8	NP	NP	54.4	5.2	9.6	29	Longitudinal study; 2ndear	M	Nor	Andersen et al. (1976) (27)

9.5	0.3	9	33.4	6.2	139	6.0	17.2	2.4	42.3	6.9	16.3	269	European Youth Heart Study	M	Eur	Ortega et al. (2010) (28)
9.5	0.4	NP	32.4	5.8	138	6.0	16.9	2.1	42.5	6.0	14.1	347	European Youth Heart Study	M	Eur	Ortega et al. (2010) (28)
9.6	0.9	8-10	NP	NP	NP	NP	NP	NP	42.6	8.3	19.5	279	20% overweight; 21% obese	P	Can	Herman et al. (2015) (29)
9.8	0.5	8-11	35.8	7.4	142	6.0	NP	NP	52.0	8.1	15.6	177	VO_{2max} = 45.1-54.6	P	Fin	Agbaje et al. (2019) (30)
9.9	0.6	8.6-11.1	34.4	6.9	141	6.5	17.4	2.6	42.0	7.0	16.7	127	VO_{2max} =22.0-58.3	P	Sca	Dencker et al. (2006) (31)
9.9	0.6	NP	34.4	6.9	140	6.5	17.4	2.6	42.0	7.0	16.7	113	Baseline	P	Swe	Danielson et al (2018) (32)
9.9	2.3	6-17	34.4	10.6	141	13.6	NP	NP	46.3	6.8	14.7	70		M	Ger	Foraita et al. (2015) (33)
9.9	0.4	NP	32.2	3.4	141	4.6	NP	NP	46.7	6.1	13.1	28	BMI in categories; most were "normal"	M	Nor	Andersen & Ghesquiere (1972) (34)
9.9	NP	9.5-10.1	35.6	NP	142	11.2	NP	NP	51.9	15.5	29.9	71	Part of the PANIC study	P	Fin	Lintu et al. (2015) (35)
9.9	0.3	NP	32.0	4.0	138	5.0	NP	NP	47.7	7.4	15.5	31		P	UK	McNarry et al. (2015) (36)
10	NP	9-10	26.7	NP	134	NP	14.8	NP	44.6	NP		87	VO_{2max} = 43.3-45.8; MWCE	M	Tan	Aandstad et al. (2006) (37)
10	NP	9-10	33.5	NP	140	NP	17.0	NP	45.9	NP		195	VO_{2max} = 44.9-46.9; MWCE	M	Nor	Aandstad et al. (2006) (37)
10.4	NP	10	33.9	5.0	141	5.5	NP	NP	60.0	6.5	10.8	31	Year 3 of a longitudinal study	M	Nor	Rutenfranz et al. (1981) (38)
10.5	0.5	10-11	37.2	6.6	141	4.7	NP	NP	46.1	6.0	13.0	50	Control group	M	Fra	Mandigout et al. (2001) (39)
10.6	0.3	NP	36.3	6.2	143	7.0	NP	NP	47.4	5.5	11.6	25		P	UK	Fawkner & Armstrong (2004) (40)
10.7	0.4	NP	36.0	5.0	141	5.0	NP	NP	53.7	6.1	11.4	48	Low altitude, high SES	M	Bol	Fellmann et al. (1994) (41)
10.7	0.8	NP	31.0	4.0	132	4.0	NP	NP	51.6	5.9	11.4	30	Low altitude, low SES	M	Bol	Fellmann et al. (1994) (41)
10.7	0.4	10-11	37.3	7.5	143	6.1	NP	NP	47.2	7.9	16.7	35	Training group (pre)	M	Fra	Mandigout et al. (2001) (39)
10.8	0.4	NP	34.9	5.2	146	6.0	NP	NP	53.4	5.3	9.9	45		M	Aus	Gaisl & Buchberger (1980) (42)
10.8	NP	9-10	35.2	5.9	142	6.7	NP	NP	48.8	8.8	18.0	62		M	Can	Cunningham et al. (1984) (43)
10.9	0.7	NP	30.0	4.0	131	5.0	NP	NP	46.7	7.5	16.1	44	High altitude, low SES	M	Bol	Fellmann et al. (1994) (41)
11.0	1.0	NP	37.0	8.0	144	7.0	NP	NP	45.7	9.3	20.4	25		M		Boissiere et al. (2013) (44)
8.83	0.93	5-9	32.2	6.0	138	7.0	16.9	2.1	45.86	5.94		40	excluded BMI over 30 and athletes	M	Fra/ Net/ Bel	Van der Steeg 2021

7.4	0.3	7	26.3	4.4	126	5.1	NP	NP	47.0	5.8	12.3	18	Test of a field procedure	M	Fra	Van Praagh et al. (1988) (45)
7.7	0.3	NP	27.4	3.4	130	4.4	NP	NP	55.5	7.0	12.6	18		M	Fin	Tompuri et al. (2015) (46)
8.2	NP	8	NP	NP	NP	NP	NP	NP	49.0	9.2	18.8	21		M	Nor	Rutenfranz et al. (1990) (47)
8.7	NP	8.3-9.8	27.6	3.6	132	6.1	NP	NP	52.7	6.3	12.0	20		M	Swe	Sunnegardh & Bratteby (1987) (48)
9	NP	8-10	26.2	2.8	130	4.4	NP	NP	50.0	6.5	13.0	19	Used IBP Handbook (1969)	M	Jap	Ikai & Kitagawa (1972) (49)
9.7	0.7		45.6	6.3	141	5.4	22.7	2.5	33.8	4.7	13.9	16	Obese prepubertal boys	P	Fra	Zunquin et al. (2009) (50)
10	NP	10	33.3	6.6	139	7.4	NP	NP	55.4	6.2	11.2	20		M	Can	Massicotte et al. (1985) (51)
10	NP	10	NP	NP	NP	NP	NP	NP	44.2	3.8	8.6	15	Rural boys	M	Jap	Yoshizawa (1972) (52)
10	NP	10	35.2	6.7	142	6.0	NP	NP	47.4	7.0	14.8	20	Have PE class 8 times/week	M	Aut	Petzl et al. (1988) (53)
10.2	NP	10	NP	NP	NP	NP	NP	NP	50.5	10.4	20.6	19		M	Nor	Rutenfranz et al. (1990) (47)
10.3	0.8	9-12	32.9	5.4	141	6.2	16.2	1.3	38.4	3.8	9.9	20	Normal-weight parents	P	Isr	Weinstein et al. (2004) (54)
10.3	0.8	9-12	31.5	3.1	140	5.5	16	1.1	40.4	4.9	12.1	20	One obese parent	P	Isr	Weinstein et al. (2004) (54)
10.3	0.9	NP	36.9	9.1	142	7.1	NP	NP	43.5	5.2	12.0	15		M	Fra	Leclair et al. (2011) (55)
10.3	0.8	NP	32.9	2.0	138	2.0	17.1	0.7	40.0	2.1	5.3	18	Lean subjects	P	Tha	Chuensiri et al. (2015) (56)
10.3	0.9	NP	38.9	10.2	142	7.0	NP	NP	43.8	5.2	11.9	15	VO_{2max} = 38.0-58.9	P	Fra	Leclair et al. (2013) (57)
10.3	0.2	8-12	NP	NP	NP	NP	NP	NP	51.4	1.1	2.1	16	Trained: test of exercise-induced hypoxia	M	Fra	Nourry et al. (2004) (58)
10.4	0.2	NP	57.9	3.0	150	3.0	25.4	3.3	30.8	1.9	6.2	17	Obese subjects	P	Tha	Chuensiri et al. (2015) (56)
10.5	0.4	NP	36.3	7.2	140	4.0	NP	NP	44.7	6.5	14.5	15	Control group	P	H-K	McManus (2005) (59)
10.5	NP	10	36.0	NP	NP	NP	NP	NP	54.3	NP		20	VO_{2max} =46-62 mL/kg-min	M	Nor	Hermansen & Oseid (1971) (60)
10.6	0.3	NP	37.9	5.4	144	4.6	NP	NP	47.9	7.0	14.6	17		M	Fra	Vinet et al. (2003) (61)
10.6	0.3	NP	35.5	5.4	141	5.6	NP	NP	56.6	7.7	13.6	15	Competitive hockey players	M	Can	Cunningham et al. (1976) (62)
10.8	0.5	NP	37.6	7.4	148	7.3	NP	NP	54.4	7.6	14.0	18		M	Can	Docherty & Gaul (1991) (63)
10.9	1.0	NP	33.2	5.2	142	6.0	NP	NP	54.0	9.7	18.0	16		M	Isr	Falk & Bar-Or (1993) (64)

11	NP	10-11	30.8	4.1	135	4.2	NP	NP	49.1	7.9	16.1	18		M	Jap	Ikai & Kitagawa (1972) (49)
11	0.7	NP	37.0	9.0	140	7.0	NP	NP	47.4	6.3	13.3	23	High altitude, high SES	M	Bol	Fellmann et al. (1994) (41)
8.4	NP	7-9	27.2	3.3	131	5.9	NP	NP	52.6	5.5	10.5	12	Non-active in sports/ with a warmup	M	Isr	Inbar & Bar-Or (1975) (65)
8.4	NP	7-9	27.2	0.3	131	5.9	NP	NP	49.4	4.7	9.5	12	Inactive, without warmup period	M	Isr	Inbar & Bar-Or (1975) (65)
9	NP	9	NP	NP	NP	NP	NP	NP	42.9	5.9	13.8	8	Rural boys	M	Jap	Yoshizawa (1972) (52)
9.3	0.3	8-10	27.1	1.3	NP	NP	NP	NP	52.8	2.1	4.0	9	Exercise in heated space	M	Isr	Inbar et al. (1981) (66)
9.5	0.3	9	34.7	6.2	141	6.3	17.4	2.5	50.3	6.8	13.5	10	Test of new VO_{2peak} equation	P	Ice	Arngrímsson et al. (2008) (67)
9.5	1.1	8-12	47.9	13.8	148	9.1	21.7	4.7	36.4	6.2	17.0	9	Overweight group	P	Aus	Crisp et al. (2012) (68)
9.7	1.0	NP	31.6	5.4	142	8.3	15.5	1.9	52.4	11.4	21.8	9	Normal weight group	P	Aus	Crisp et al. (2012) (68)
9.7	0.3	8-10	32.2	4.8	NP	NP	NP	NP	54.1	6.6	12.2	9	Exercise in air conditioning	M	Isr	Inbar et al. (1981) (66)
9.9	0.6	NP	41.7	13.9	140	5.7	20.9	5.9	39.2	7.8	19.9	12	42% overweight/obese	M	Chi	Garcia-Prieto et al. (2017) (69)
9.9	1.6	8-12	52.9	13.9	141	11.0	26.1	3.5	32.8	4.0	12.2	7	Obese subjects; pre-exercise program	M	Ita	Lazzer et al. (2008) (70)
10	NP	10	NP	NP	NP	NP	NP	NP	45.1	2.3	5.1	5	Urban boys	M	Jap	Yoshizawa (1972) (52)
10	NP	10	36.4	1.7	139	8.0	NP	NP	47.1	10.2	21.7	11	Have PE class 3 times/week	M	Aut	Petzl et al. (1988) (53)
10.2	NP	10	28.7	3.0	132	2.2	NP	NP	38.6	4.6	11.9	8		M	Jap	Yamaji & Miyashita (1977) (71)
10.2	0.4	NP	32.4	3.5	142	5.1	NP	NP	56.4	4.6	8.2	10	Second experiment	P	Den	Hansen et al. (1989) (72)
10.3	0.1	10-11	38.9	5.6	145	4.0	NP	NP	45.5	3.4	7.5	10	Interval training group (pre-training)	P	H-K	McManus (2005) (59)
10.3	NP	NP	33.2	5.7	143	7.8	NP	NP	49.3	6.7	13.6	14	Part of a longitudinal study	P	Nor	Andersen et al. (1974) (22)
10.4	0.4	NP	35.9	7.3	140	4.0	NP	NP	47.0	6.5	13.8	10	Continuous training group (pre-training)	P	H-K	McManus (2005) (59)
10.5	0.7	NP	51.0	9.0	146	5.2	24	3.4	51.5	7.5	14.6	14	Overweight boys	P	Can	Peralta-Huertas et al. (2008) (73)
10.5	0.3	10-11	37.5	5.4	142	5.0	NP	NP	44.1	6.1	13.8	9	Endurance training group (pre)	M	Fra	Obert et al. (2003) (74)
10.5	0.3	10-11	36.3	7.9	144	7.1	NP	NP	46.2	6.7	14.5	9	First experiment	M	Den	Hansen et al. (1989) (72)
10.5	1.1	NP	32.9	5.1	142	7.5	16.3	1.10	49.0	7.9	16.1	12	Inactive boys	M	Fra	Birat et al. (2018) (75)

10.5	0.8	NP	34.8	6.0	142	7.4	NP	NP	43.5	5.4	12.4	8		P	Isr	Dotan et al. (2000) (76)
10.6	0.9	NP	34.7	6.9	143	7.1	NP	NP	46.6	NP		12	Training group (pre-experiment)	P	UK	Tolfrey et al. (1998) (77)
10.6	0.3	10-11	36.9	6.1	143	5.0	NP	NP	51.5	6.3	12.2	9	Control group (pre)	M	Fra	Obert et al. (2003) (74)
10.6	1.0	NP	35.0	5.0	142	10.0	NP	NP	48.0	8.0	16.7	10		M	Fra	Martin et al. (2002) (78)
10.7	0.9	NP	35.7	6.6	147	8.4	NP	NP	48.0	6.0	12.5	9		P	Bel	Vandekerckhove et al. (2016) (79)
11	NP	10-11	35.3	8.1	141	5.5	17.6	3.2	50.1	12.4	24.8	12	Deaf boys	M	Pol	Zebrowska & Zwierzchowska (2009) (80)
11	NP	10-11	33.4	7.2	143	8.4	16.3	2.3	52.9	12.2	23.1	12	Normal hearing boys	M	Pol	Zebrowska & Zwierzchowska (2009) (80)
8.1	0.3	7-10	27.8	1.7	132	1.7	15.8	0.6	47.3	5.00		12	healthy boys	P	Ger	Schöffl et al. (2020) (81)
10.7	0.8	9-11	43.4	8.6	146.2	4.5	20.2	3.1	43.0	3.6		11	healthy boys	P	Bra	Langer et al. 2020 (82)
9.6	2.8	7-11	36.2	6.8	140	8.7	17.7	4.8	46.3	10.20		14			Fin	Haapala et al. 2021 (83)

(Y=years, VO_{2max}=maximal oxygen consumption, NP=not provided, BSA=body surface area, LBM=lean body mass, PR=Puerto Rico , Fra=France, Spa=Spain, Nor=Norway, Eur=Europe, Can=Canada, Fin=Finland, Sca=Scandinavian countries, Swe=Sweden, Ger=Germany, Tan=Tanzania, Bol=Bolivia, Aus=Australia, Net*Netherlands, Bel=Belgium, Jap=Japan, Aut=Austria, Isr=Israel, H-K=Hong Kong, Ice=Iceland, Chi=China, Ita=Italy, Den=Denmark, Pol=Poland, Bra=Brasil)

References

1. Springer C, Barstow TJ, Wasserman K, Cooper DM. Oxygen uptake and heart rate responses during hypoxic exercise in children and adults. *Med Sci Sports Exerc*. 1991;23(1):71–9.

2. Washington RL, van Gundy JC, Cohen C, Sondheimer HM, Wolfe RR. Normal aerobic and anaerobic exercise data for North American

school-age children. *J Pediatr*. 1988;112(2):223–33.

3. McMurray RG, Harrell JS, Bradley C, Deng S, Bangdiwala SI. Predicted maximal aerobic power inouth is related to gender, ethnicity, and body composition. *Med Sci Sport Exerc*. 2001;33(5):S31.

4. Sockolov R, Irwin B, Dressendorfer RH, Bernauer EM. Exercise performance in 6 to 11ear old boys with Duchenne muscular dystrophy. *Arch Phys Med Rehabil*. 1977;58(5):195–201.

5. Rivera-Brown AM, Alvarez M, Rodríguez-Santana JR, Benetti PJ. Anaerobic power and achievement of $\dot{V}O2$ plateau in pre-pubertal boys. *Int J Sports Med*. 2001;22(2):111–5.

6. Turley KR, Wilmore JH. Cardiovascular responses to submaximal exercise in 7- to 9-yr-old boys and girls. *Med Sci Sports Exerc*. 1997;29(6):824–32.

7. Gilliam TB, Katch VL, Thorland W, Weltman A. Prevalence of coronary heart disease risk factors in active children, 7 to 12ears of age. *Med Sci Sports Exerc*. 1977;9(1):21–5.

8. Barkley JE, Roemmich JN. Validity of the CALER and OMNI-bike ratings of perceived exertion. *Med Sci Sports Exerc*. 2008;40(4):760–6.

9. Swain KE, Rosenkranz SK, Beckman B, Harms CA. Expiratory flow limitation during exercise in prepubescent boys and girls: prevalence

and implications. *J Appl Physiol*. 2010;108(5):1267–74.

10. Robertson RJ, Goss FL, Boer N, et al. OMNI scale perceived exertion at ventilatory breakpoint in children: Response normalized. *Med Sci Sports Exerc*. 2001;33(11):1946–52.

11. Cooper DM, Weiler-Ravell D, Whipp BJ, Wasserman K. Aerobic parameters of exercise as a function of body size during growth in children. *J Appl Physiol Respir Environ Exerc Physiol*. 1984;56(3):628–34.

12. Rivas E, Huynh H, Galassetti PR. Obesity Affects Submaximal Oxygen Uptake-Heart Rate Relationship and Exercise Economy Differently in Pre- and Post-pubescent Boys and Girls. *Int J Exerc Sci*. 2019;12(3):748–63.

13. Mahon AD, Anderson CS, Hipp MJ, Hunt KA. Heart Rate Recovery from Submaximal Exercise in Boys and Girls. *Med Sci Sports Exerc*. 2003;35(12):2093–7.

14. Rowland TW, Rambusch JM, Staab JS, Unnithan VB, Siconolfi SF. Accuracy of physical working capacity (PWC170) in estimating aerobic fitness in children. *J Sports Med Phys Fitness*. 1993;33(2):184–8.

15. Janz KF, Burns TL, Witt JD, Mahoney LT. Longitudinal analysis of scaling $\dot{V}O2$ for differences in body size during puberty: The muscatine study. *Med Sci Sports Exerc*. 1998;30(9):1436–44.

16. Del Corral P, Mahon AD, Duncan GE, Howe CA, Craig BW. The effect of exercise on serum and salivary cortisol in male children. *Med*

Sci Sports Exerc. 1994;26(11):1297–301.

17. Janz KF, Dawson JD, Mahoney LT. Tracking physical fitness and physical activity from childhood to adolescence: The Muscatine study. *Med Sci Sports Exerc*. 2000;32(7):1250–7.

18. Rowland TW. *The development of aerobic fitness in children*. London: E & FN Spon.; 1997. 179–189 p.

19. DeStefano RA, Caprio S, Fahey JT, Tamborlane W V, Goldberg B. Changes in body composition after a 12-wk aerobic exercise program in obese boys. *Pediatr Diabetes*. 2000;1(2):61–5.

20. Falgairette G, Bedu M, Fellmann N, Van-Praagh E, Coudert J. Bio-energetic profile in 144 boys aged from 6 to 15ears with special reference to sexual maturation. *Eur J Appl Physiol Occup Physiol*. 1991;62(3):151–6.

21. Guerrero L, Naranjo J, Carranza MD. Influence of gender on ventilatory efficiency during exercise inoung children. *J Sports Sci*. 2008;26(13):1455–7.

22. Lange Andersen K, Seliger V, Rutenfranz J, Mocellin R. Physical performance capacity of children in Norway - Part I. Population parameters in a rural inland community with regard to maximal aerobic power. *Eur J Appl Physiol Occup Physiol*. 1974;33(3):177–95.

23. Steene-Johannessen J, Anderssen SA, Kolle E, Andersen LB. Low muscle fitness is associated with metabolic risk inouth. *Med Sci Sports Exerc*. 2009;41(7):1361–7.

24. Wedderkopp N, Froberg K, Hansen HS, Riddoch C, Andersen LB. Cardiovascular Risk Factors Cluster in Children and Adolescents with Low Physical Fitness: The European Youth Heart Study (EYHS). *Pediatr Exerc Sci*. 2003;15(4):419–27.

25. Kolle E, Steene-Johannessen J, Andersen LB, Anderssen SA. Objectively assessed physical activity and aerobic fitness in a population-based sample of Norwegian 9- and 15-year-olds. *Scand J Med Sci Sport* [Internet]. 2010 [cited 2022 Jun 30];20(1) Available from: https://pubmed.ncbi.nlm.nih.gov/19422647/. doi:10.1111/j.1600-0838.2009.00892.x.

26. Adegboye ARA, Anderssen SA, Froberg K, et al. Recommended aerobic fitness level for metabolic health in children and adolescents: A study of diagnostic accuracy. *Br J Sports Med*. 2011;45(9):722–8.

27. Lange Andersen K, Seliger V, Rutenfranz J, Skrobak-Kaczynski J. Physical performance capacity of children in Norway - Part IV. The rate of growth in maximal aerobic power and the influence of improved physical education of children in a rural community - population parameters in a rural community. *Eur J Appl Physiol Occup Physiol*. 1976;35(1):49–58.

28. Ortega FB, Ruiz JR, Hurtig-Wennlöf A, et al. Cardiovascular fitness modifies the associations between physical activity and abdominal adiposity in children and adolescents: The Europeanouth heart study. *Br J Sports Med*. 2010;44(4):256–62.

29. Herman KM, Chaput JP, Sabiston CM, Mathieu ME, Tremblay A, Paradis G. Combined physical activity/sedentary behavior associations with indices of adiposity in 8- to 10-year-old children. *J Phys Act Heal*. 2015;12(1):20–9.

30. Agbaje AO, Haapala EA, Lintu N, et al. Peak oxygen uptake cut-points to identify children at increased cardiometabolic risk – The

PANIC Study. *Scand J Med Sci Sport*. 2019;29(1):16–24.

31. Dencker M, Thorsson O, Karlsson MK, et al. Daily physical activity and its relation to aerobic fitness in children aged 8-11ears. *Eur J Appl Physiol*. 2006;96(5):587–92.

32. Danielson A, Thorsson O, Karlsson MK, Wollmer P, Andersen LB, Dencker M. School-based study found that physical activity and aerobic fitness predicted increases in total body fat and abdominal fat at a mean age of 9.8ears. *Acta Paediatr Int J Paediatr*. 2018;107(10):1810–7.

33. Foraita R, Brandes M, Günther F, Bammann K, Pigeot I, Ahrens W. The influence of aerobic fitness on obesity and its parent-offspring correlations in a cross-sectional study among German families. *BMC Public Health* [Internet]. 2015 [cited 2022 Jun 30];15(1) Available from: https://pubmed.ncbi.nlm.nih.gov/26162746/. doi:10.1186/s12889-015-2013-x.

34. Andersen KL, Ghesquiere J. Sex differences in maximal oxygen uptake, heart rate and oxygen pulse at 10 and 14ears in Norwegian children. *Hum Biol*. 1972;44(3):413–31.

35. Lintu N, Viitasalo A, Tompuri T, et al. Cardiorespiratory fitness, respiratory function and hemodynamic responses to maximal cycle ergometer exercise test in girls and boys aged 9–11ears: the PANIC Study. *Eur J Appl Physiol*. 2015;115(2):235–43.

36. McNarry MA, Farr C, Middlebrooke A, et al. Aerobic Function and Muscle Deoxygenation Dynamics during Ramp Exercise in Children. *Med Sci Sports Exerc*. 2015;47(9):1877–84.

37. Aandstad A, Berntsen S, Hageberg R, Klasson-Heggebø L, Anderssen SA. A comparison of estimated maximal oxygen uptake in 9 and 10ear old schoolchildren in Tanzania and Norway. *Br J Sports Med*. 2006;40(4):287–92.

38. Rutenfranz J, Andersen KL, Seliger V, Klimmer F, Berndt I, Ruppel M. Maximum aerobic power and body composition during the puberty growth period: Similarities and differences between children of two European countries. *Eur J Pediatr*. 1981;136(2):123–33.

39. S M, AM L, D C, P G, P O. Effect of gender in response to an aerobic training programme in prepubertal children. *Acta Paediatr*. 2001;90(1):9–15.

40. Fawkner SG, Armstrong N. Sex differences in the oxygen uptake kinetic response to heavy-intensity exercise in prepubertal children. *Eur J Appl Physiol*. 2004;93(1–2):210–6.

41. Fellmann N, Beaune B, Coudert J. Blood lactate after maximal and supramaximal exercise in 10- to 12-year-old Bolivian boys effects of altitude and socioeconomic status. *Int J Sports Med* [Internet]. 1994 [cited 2022 Jun 30];15(SUPPL. 2) Available from: https://pubmed.ncbi.nlm.nih.gov/8056467/. doi:10.1055/s-2007-1021119.

42. Gaisl G, Buchberger J. *Determination of the aerobic and anaerobic thresholds of 10-11-year-old boys using blood-gas analysis*. Baltimore: University Park Press; 1980. 93–98 p.

43. Cunningham DA, Paterson DH, Blimkie CJR, Donner AP. Development of cardiorespiratory function in circumpubertal boys: A longitudinal study. *J Appl Physiol Respir Environ Exerc Physiol*. 1984;56(2):302–7.

44. Boissière J, Maufrais C, Baquet G, et al. Specific left ventricular twist-untwist mechanics during exercise in children. *J Am Soc Echocardiogr*. 2013;26(11):1298–305.

45. Van Praagh E, Bedu M, Falgairette G, Fellmann N, Coudert J. Comparaison entre over VO2 max direct et indirect chez l'enfant de 7 et 12 ans. Validation d'une épreuve de terrain. *Sci Sport*. 1988;3(4):327–32.

46. Tompuri T, Lintu N, Savonen K, et al. Measures of cardiorespiratory fitness in relation to measures of body size and composition among children. *Clin Physiol Funct Imaging*. 2015;35(6):469–77.

47. Rutenfranz J, Máček M, Lange Andersen K, et al. The relationship between changing body height and growth related changes in maximal aerobic power. *Eur J Appl Physiol Occup Physiol*. 1990;60(4):282–7.

48. Sunnegårdh J, Bratteby LE. Maximal oxygen uptake, anthropometry and physical activity in a randomly selected sample of 8 and 13ear old children in Sweden. *Eur J Appl Physiol Occup Physiol*. 1987;56(3):266–72.

49. Ikai M, Kitagawa K. Maximum oxygen uptake of japanese related to sex and age. *Med Sci Sports Exerc*. 1972;4(3):127–31.

50. Zunquin G, Theunynck D, Sesboüé B, Arhan P, Bouglé D. Evolution of fat oxidation during exercise in obese pubertal boys: Clinical implications. *J Sports Sci*. 2009;27(4):315–8.

51. Massicotte DR, Gauthier R, Markon P. Prediction of VO2max from the running performance in children aged 10-17ears. *J Sports Med*

Phys Fitness. 1985;25(1–2):10–7.

52. Yoshizawa S. A comparative study of aerobic work capacity in urban and rural adolescents. *J Hum Ergol (Tokyo)*. 1972;1(1):45–65.

53. Petzl DH, Haber P, Schuster E, Popow C, Haschke F. Reliability of estimation of maximum performance capacity on the basis of submaximum ergometric stress tests in children 10-14ears old. *Eur J Pediatr*. 1988;147(2):174–8.

54. Weinstein Y, Kamerman T, Berry E, Falk B. Mechanical Efficiency of Normal-Weight Prepubertal Boys Predisposed to Obesity. *Med Sci Sports Exerc*. 2004;36(4):567–73.

55. Leclair E, Mucci P, Borel B, Baquet G, Carter H, Berthoin S. Time to exhaustion and time spent at a high percentage of Vo2max in severe intensity domain in children and adults. *J Strength Cond Res*. 2011;25(4):1151–8.

56. Chuensiri N, Tanaka H, Suksom D. The acute effects of supramaximal high-intensity intermittent exercise on vascular function in Lean vs. Obese prepubescent boys. *Pediatr Exerc Sci*. 2015;27(4):503–9.

57. Leclair E, Berthoin S, Borel B, et al. Faster pulmonary oxygen uptake kinetics in children vs adults due to enhancements in oxygen delivery and extraction. *Scand J Med Sci Sport*. 2013;23(6):705–12.

58. Nourry C, Fabre C, Bart F, Grosbois JM, Berthoin S, Mucci P. Evidence of Exercise-Induced Arterial Hypoxemia in Prepubescent Trained Children. *Pediatr Res*. 2004;55(4):674–81.

59. McManus AM, Cheng CH, Leung MP, Yung TC, Macfarlane DJ. Improving aerobic power in primary school boys: A comparison of continuous and interval training. *Int J Sports Med*. 2005;26(9):781–6.

60. Hermansen L, Oseid S. Direct and indirect estimation of maximal oxygen uptake in pre-pubertal boys. *Acta Paediatr Scand Suppl*. 1971;217:18–23.

61. Vinet A, Mandigout S, Nottin S, et al. Influence of Body Composition, Hemoglobin Concentration, and Cardiac Size and Function of Gender Differences in Maximal Oxygen Uptake in Prepubertal Children. *Chest*. 2003;124(4):1494–9.

62. Cunningham DA, Telford P, Swart GT. The cardiopulmonary capacities ofoung hockey players: Age 10. *Med Sci Sports Exerc*. 1976;8(1):23–5.

63. Docherty D, Gaul CA. Relationship of body size, physique, and composition to physical performance inoung boys and girls. *Int J Sports Med*. 1991;12(6):525–32.

64. Falk B, Bar-Or O. Longitudinal Changes in Peak Aerobic and Anaerobic Mechanical Power of Circumpubertal Boys. *Pediatr Exerc Sci*. 1993;5(4):318–31.

65. Inbar O, Bar-Or O. The effects of intermittent warm-up on 7-9ear-old boys. *Eur J Appl Physiol Occup Physiol*. 1975;34(1):81–9.

66. Inbar O, Bar-Or O, Dotan R, Gutin B. Conditioning versus exercise in heat as methods for acclimatizing 8- to 10-yr-old boys to dry heat. *J*

Appl Physiol Respir Environ Exerc Physiol. 1981;50(2):406–11.

67. Arngrímsson SÁ, Sveinsson T, Jóhannsson E. Peak oxygen uptake in children: Evaluation of an older prediction method and development of a new one. *Pediatr Exerc Sci*. 2008;20(1):62–73.

68. Crisp NA, Guelfi KJ, Licari MK, Braham R, Fournier PA. Does exercise duration affect Fat max in overweight boys? *Eur J Appl Physiol*. 2012;112(7):2557–64.

69. García-Prieto JC, Martinez-Vizcaino V, García-Hermoso A, et al. Energy expenditure in playground games in primary school children measured by accelerometer and heart rate monitors. *Int J Sport Nutr Exerc Metab*. 2017;27(5):467–74.

70. Lazzer S, Molin M, Stramare D, Facchini S, Francescato MP. Effects of an eight-month weight-control program on body composition and lipid oxidation rate during exercise in obese children. *J Endocrinol Invest*. 2008;31(6):509–14.

71. Yamaji K, Miyashita M. Oxygen transport system during exhaustive exercise in Japanese boys. *Eur J Appl Physiol Occup Physiol*. 1977;36(2):93–9.

72. Hansen HS, Froberg K, Nielsen JR, Hyldebrandt N. A new approach to assessing maximal aerobic power in children: the Odense School Child Study. *Eur J Appl Physiol Occup Physiol*. 1989;58(6):618–24.

73. Peralta-Huertas J, Livingstone K, Banach A, Klentrou P, O'Leary D. Differences in left ventricular mass between overweight and normal-

weight preadolescent children. *Appl Physiol Nutr Metab*. 2008;33(6):1172–80.

74. Obert P, Mandigouts S, Nottin S, Vinet A, N'Guyen LD, Lecoq AM. Cardiovascular responses to endurance training in children: Effect of gender. *Eur J Clin Invest*. 2003;33(3):199–208.

75. Birat A, Bourdier P, Piponnier E, et al. Metabolic and fatigue profiles are comparable between prepubertal children and well-trained adult endurance athletes. *Front Physiol* [Internet]. 2018 [cited 2022 Jun 30];9(APR) Available from: https://pubmed.ncbi.nlm.nih.gov/29740332/. doi:10.3389/fphys.2018.00387.

76. Dotan R, Falk B, Raz A. Intensity effect of active recovery from glycolytic exercise on decreasing blood lactate concentration in prepubertal children. *Med Sci Sports Exerc*. 2000;32(3):564–70.

77. Tolfrey K, Campbell IG, Batterham AM. Aerobic trainability of prepubertal boys and girls. *Pediatr Excercise Sci*. 1998;10(3):248–63.

78. Martin R, Hautier C, Bedu M. Effect of age and pedalling rate on cycling efficiency and internal power in humans. *Eur J Appl Physiol*. 2002;86(3):245–50.

79. Vandekerckhove K, Coomans I, Moerman A, De Wolf D, Boone J. Characterizing cerebral and locomotor muscle oxygenation to incremental ramp exercise in healthy children: relationship with pulmonary gas exchange. *Eur J Appl Physiol*. 2016;116(11–12):2345–55.

80. Zebrowska A, Zwierzchowska A. Spirometric values and aerobic efficency of children and adolescents with hearing loss. *J Physiol*

Pharmacol. 2006;57(SUPPL. 4):443–7.

81. Schöffl I, Ehrlich B, Stanger S, Rottermann K, Dittrich S, Schöffl V. Exercise Field Testing in Children: A New Approach for Age-Appropriate Evaluation of Cardiopulmonary Function. *Pediatr Cardiol*. 2020;41(6):1099–106.

82. Langer RD, da Costa KG, Bortolotti H, Fernandes GA, de Jesus RS, Gonçalves EM. Phase angle is associated with cardiorespiratory fitness and body composition in children aged between 9 and 11ears. *Physiol Behav* [Internet]. 2020 [cited 2022 May 28];215 Available from: https://pubmed.ncbi.nlm.nih.gov/31837386/. doi:10.1016/j.physbeh.2019.112772.

83. Haapala EA, Gao Y, Hartikainen J, Rantalainen T, Finni T. Associations of fitness, motor competence, and adiposity with the indicators of physical activity intensity during different physical activities in children. *Sci Rep* [Internet]. 2021 [cited 2022 May 28];11(1) Available from: https://pubmed.ncbi.nlm.nih.gov/34131258/. doi:10.1038/s41598-021-92040-2.
